# Tc17 Cells Mediate Vaccine Immunity against Lethal Fungal Pneumonia in Immune Deficient Hosts Lacking CD4^+^ T Cells

**DOI:** 10.1371/journal.ppat.1002771

**Published:** 2012-07-19

**Authors:** Som Gowda Nanjappa, Erika Heninger, Marcel Wüthrich, David Joseph Gasper, Bruce S. Klein

**Affiliations:** 1 The Department of Pediatrics, School of Medicine and Public Health, University of Wisconsin Madison, Madison, Wisconsin, United States of America; 2 The Department of Pathobiological Sciences, School of Veterinary Medicine, University of Wisconsin Madison, Madison, Wisconsin, United States of America; 3 The Department of Internal Medicine, School of Medicine and Public Health, University of Wisconsin Madison, Madison, Wisconsin, United States of America; 4 The Department of Medical Microbiology and Immunology, School of Medicine and Public Health, University of Wisconsin Madison, Madison, Wisconsin, United States of America; University of Massachusetts Medical School, United States of America

## Abstract

Vaccines may help reduce the growing incidence of fungal infections in immune-suppressed patients. We have found that, even in the absence of CD4^+^ T-cell help, vaccine-induced CD8^+^ T cells persist and confer resistance against *Blastomyces dermatitidis* and *Histoplasma capsulatum*. Type 1 cytokines contribute to that resistance, but they also are dispensable. Although the role of T helper 17 cells in immunity to fungi is debated, IL-17 producing CD8^+^ T cells (Tc17 cells) have not been investigated. Here, we show that Tc17 cells are indispensable in antifungal vaccine immunity in hosts lacking CD4^+^ T cells. Tc17 cells are induced upon vaccination, recruited to the lung on pulmonary infection, and act non-redundantly in mediating protection in a manner that requires neutrophils. Tc17 cells did not influence type I immunity, nor did the lack of IL-12 signaling augment Tc17 cells, indicating a distinct lineage and function. IL-6 was required for Tc17 differentiation and immunity, but IL-1R1 and Dectin-1 signaling was unexpectedly dispensable. Tc17 cells expressed surface CXCR3 and CCR6, but only the latter was essential in recruitment to the lung. Although IL-17 producing T cells are believed to be short-lived, effector Tc17 cells expressed low levels of KLRG1 and high levels of the transcription factor TCF-1, predicting their long-term survival and stem-cell like behavior. Our work has implications for designing vaccines against fungal infections in immune suppressed patients.

## Author Summary

Systemic fungal infections have emerged as a public health problem, especially for patients with suppressed immunity. At present, there are no vaccines against fungi, partly because it is hard to elicit strong immunity in immune suppressed patients. We have found however that residual elements of T cell immunity can be harnessed by vaccination even in immune suppressed hosts. We show here that immune suppressed mice lacking T helper cells can still be vaccinated successfully against lethal fungal pneumonia. A population of T cytotoxic IL-17-producing cells (Tc17 cells) is instrumental and indispensible in vaccine protection. We describe here mechanisms that explain how these cells are induced to mature in Tc17 cells, persist for long periods in the body providing “immune memory”, recruit to the site of infection, and clear the tissue of fungi. Our work sheds new light on potent T cells that can be harnessed by vaccine strategies against fungal infection in vulnerable patients.

## Introduction

The incidence of invasive fungal infections in immune-compromised hosts has skyrocketed. These patients often have diminished or dysfunctional CD4^+^ T cells rendering them susceptible to fungal infections caused by *Candida, Aspergillus, Cryptococcus, Histoplasma* and *Pneumocystis* [1]. Thus, it would be advantageous to harness residual immunity against fungal infections in this setting. Regrettably, there are no vaccines to prevent or treat primary or opportunistic fungal infections.

Substantial progress has been made in identifying the components of innate and adaptive immunity that control mucosal and systemic fungal infections [2]–[8]. The cytokine IL-17 helps defend against mucosal infections, including those due to fungi. The product is needed for control of mucosal and cutaneous candidiasis in mice [9]. Genetic mutations in IL-17 production or signaling lead to increased susceptibility to mucocutaneous candidiasis in humans [10]. Likewise, neutralization of IL-17 enhances susceptibility to *Aspergillus* pneumonia in mice [11].

T helper (Th) 17 cells, one source of IL-17, play a prominent role in fungal infections [2], [12], [13]. Although there is controversy about the beneficial and harmful roles of IL-17 and Th17 [14]–[18], we have shown that Th17 cells are pivotal in vaccine resistance against three systemic mycoses [13]. In another study, in immune deficient mice lacking CD4^+^ T cells, vaccination against *Blastomyces* and *Histoplasma* elicited CD8^+^ T-cell immunity and resistance [19]. There, type 1 cytokines contributed significantly to resistance mediated by CD8^+^ T cells. However, individual type 1 cytokines were dispensable and compensated without any loss of resistance. The role of IL-17 producing CD8^+^ T cells (Tc17) was not explored in that study and it is unknown whether Tc17 cells can be induced without CD4^+^ T-cell help or mediate protective immunity upon fungal vaccination. Although the role of Tc17 cells in fungal infections remains unexplored, recent work shed some light on Tc17 cells during other infections. Tc17 cells lacking granzyme B have been associated with enhanced progression of SIV infection in macaques [20]. In another viral model, Tc17 cells protected against lethal influenza infection [21]. Lastly, Tc17 cells were shown to mount immunity against vaccinia virus infection by acquiring cytotoxic ability [22].

Studies of CD4^+^ T cells have provided insight into how T cells differentiate into IL-17 producing cells. Naïve CD4^+^ T cells in mice differentiate into Th17 cells with cues from IL-6 and TGFβ [23]. Differentiated Th17 cells are amplified and sustained by IL-21 and IL-23, respectively [24]. The absence of IL-6 may not abolish Th17 differentiation since IL-21, like IL-6, also can activate Stat3 [23], [25] to induce the expression of RORγt, thereby resulting in the production of IL-17 family members [26]. Additionally, pattern recognition receptor (PRR) signals augment Th17 differentiation. Dectin-1 signals suppress Th1 differentiation and promote a Th17 phenotype [27], [28]. Other C-type lectins and TLRs can augment Th17 cells [29]. The cytokine IL-1 also plays a vital role in inducing and maintaining Th17 cells [30], [31]. Th17 cells are not induced upon antigen challenge in mice lacking IL-1 [32], and IL-1 signaling in non-T cells leads to production of IL-23, which is required for maintenance of Th17 cells [31], [33]. Activation of PRR pathways also leads to production of IL-1β, which, alone or together with IL-6, IL-21 and TGFβ, enhances Th17 development [31].

To the best of our knowledge, the requisite elements of Tc17 cell differentiation during fungal infection have not been studied. Herein, we asked whether and how Tc17 cells are induced by fungal vaccination, and studied the functional role of these cells in vaccine immunity. Since we found a vital role for Tc17 cells, we investigated the PRRs and products that induce their differentiation. We also analyzed the features that might be predictive of their long-term survival and stem-cell likeness in view of our and other's recent findings [34] suggesting that IL-17 producing T cells may be longer lived than previously believed [35].

We report here that polyclonal and antigen-specific Tc17 cells are induced by fungal vaccination in the absence of CD4^+^ T-cell help. Tc17 cells were indispensable in vaccine immunity against lethal pulmonary fungal infection. Tc17 cells did not influence Tc1 cells, nor did the loss of IFN-γ producing CD8^+^ T cells abate the vaccine resistance mediated by Tc17 cells. Vaccine control of lethal fungal infection was dependent on neutrophils, and was linked functionally with CD8^+^ T-cell derived IL-17. Tc17 cells expressed both chemokine receptors CCR6 and CXCR3, but recruitment of the cells into the lung was mediated by CCR6. Vaccine-induced Tc17 cells and immunity to infection surprisingly did not require Dectin-1 or IL-1 receptor signaling, but did require IL-6. Although IL-17 producing CD4^+^ T cells are generally thought to be short-lived, Tc17 cells demonstrated a phenotype with markers that are predictive of long-term survival, multi-potency and stem-cell likeness.

## Results

### IL-17A producing CD8^+^ T cells (Tc17 cells) are primed and recalled after fungal vaccination independent of CD4^+^ T cell help

**Figure 1:**
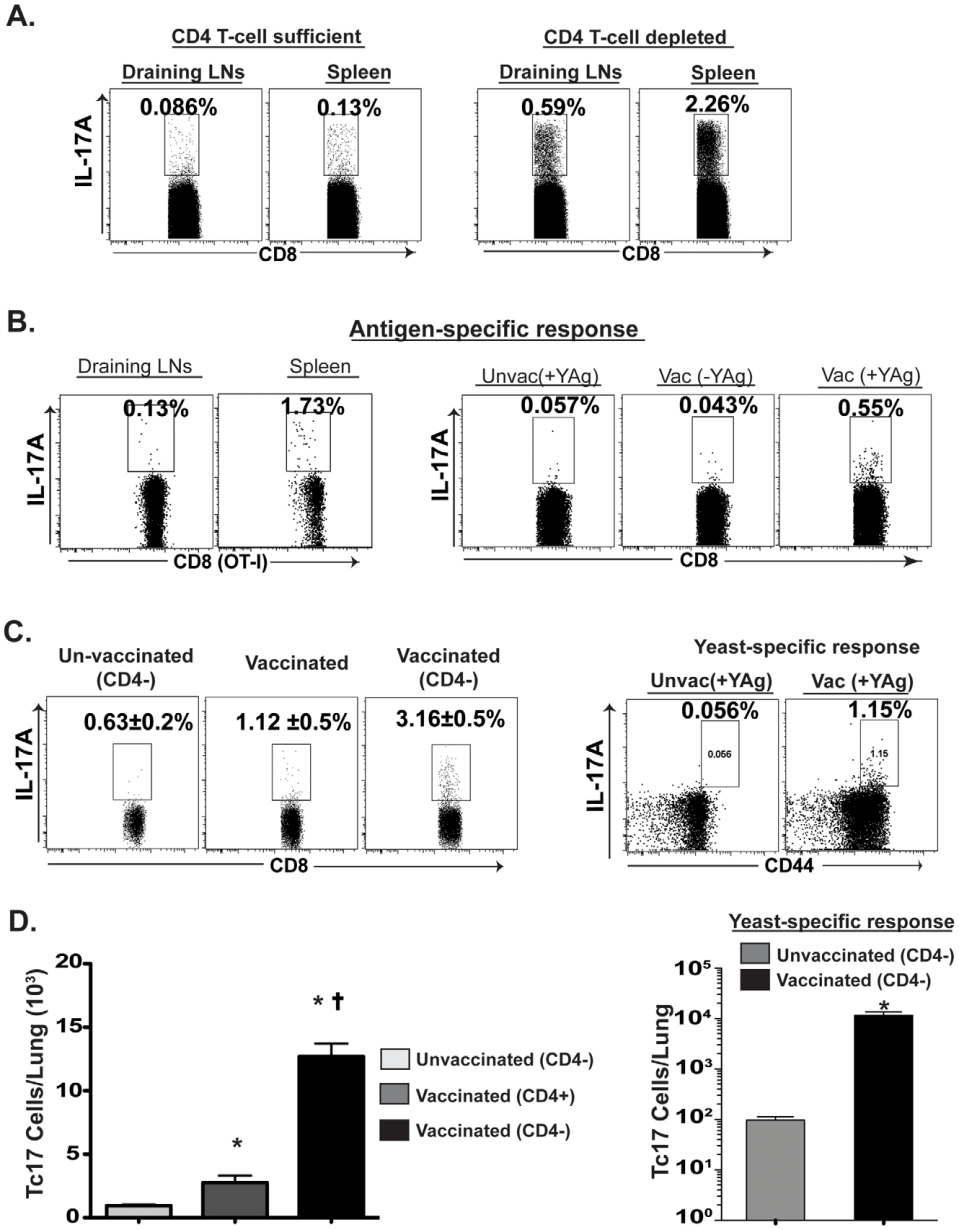
Tc17 cells are induced and recalled in the absence of CD4^+^ T cells. Mice were vaccinated as described in Methods. CD4^+^ T cells were depleted using GK1.5 mAb i.v. weekly throughout the experiment. **A.** Percent CD8^+^ T cells producing IL-17 in draining LNs (dLNs) and spleen at 19 days post-vaccination in the presence (left panels) or absence (right) of CD4^+^ T cells as assessed by flow cytometry. **B.** In the left two panels, OT-I T cells (1×10^6^) were transferred to naïve congenic CD4*^−/−^* mice and vaccinated with recombinant *Blastomyces* expressing the OT-I epitope SIINFEKL. At day 14, dLNs and spleens were harvested and stimulated *ex vivo* with OVA peptide. In the right three panels, dLNs were harvested and cultured with BMDCs in the presence or absence of heat-killed yeast. Yeast-specific OT-I and endogenous Tc17 cells were enumerated by flow cytometry. The percent (**C**) or number (**D**) of polyclonal and yeast-specific Tc17 cells during recall response in the lung 3–4 days after pulmonary infection with wild type virulent *Blastomyces*. Values are mean ± SD of 4 mice/group. * p<0.05 vs. unvaccinated mice; and ^†^ p<0.05 vs. vaccinated CD4^+^ T-cell sufficient mice.

Given that CD4^+^ T cells help in initiating the inflammation and activation of DCs needed for efficient priming of CD8^+^ T cells, we asked whether Tc17 cells are primed after vaccination without CD4^+^ T-cell help and can be recalled into the lung upon lethal pulmonary infection. The draining lymph nodes (dLNs) and spleens were analyzed for differentiation and expansion of Tc17 cells in mice that had been vaccinated with *Blastomyces* in the presence or absence of CD4^+^ T cells. After vaccination, the frequency and total number of Tc17 cells were much higher in CD4 T-cell depleted mice compared to CD4^+^ T-cell sufficient mice (p≤0.05; **Fig. 1A**).

To determine whether Tc17 cells expand in an antigen-specific manner in response to the fungal vaccine, we immunized mice with a novel recombinant vaccine strain of *Blastomyces* that expresses the model ovalbumin epitope SIINFEKL, along with adoptive transfer of OT-I cells into the mice [36]. Nearly 2% of the OT-I cells in the spleen of vaccinated CD4^+^ T-cell depleted mice were IL-17 positive (**Fig. 1B**). *In vitro* co-culture of OT-I cells similarly showed IL-17 production specifically in response to the OVA expressing vaccine strain (data not shown). To validate that Tc17 cells respond specifically to intrinsic yeast antigens, we re-stimulated dLN cells with yeast-loaded bone-marrow dendritic cells (BMDCs). CD8 T cells from vaccinated mice responded specifically to yeast antigen and were positive for IL-17A (**Fig. 1B**). We also analyzed the recall response of Tc17 cells in vaccinated mice. Tc17 cells were recruited into the lung after pulmonary challenge irrespective of CD4^+^ T-cell help (**Fig. 1C & D**). In fact, greater numbers of Tc17 cells were recruited in mice vaccinated in the absence of CD4^+^ T cells. Tc17 cells recruited to the lung responded in a *Blastomcyes* antigen-specific manner (**Fig. 1C & D**). In experiments using *Histoplasma* for vaccination, we found that Tc17 cells also were induced in response to that yeast, and recalled to the lung upon pulmonary challenge (**Fig. S1A & B**). Collectively, these data suggest that fungal-specific Tc17 cells can be induced upon fungal vaccination and recalled into the lung independent of CD4^+^ T-cell help.

### Essential role of Tc17 cells for vaccine immunity against lethal fungal infection

**Figure 2:**
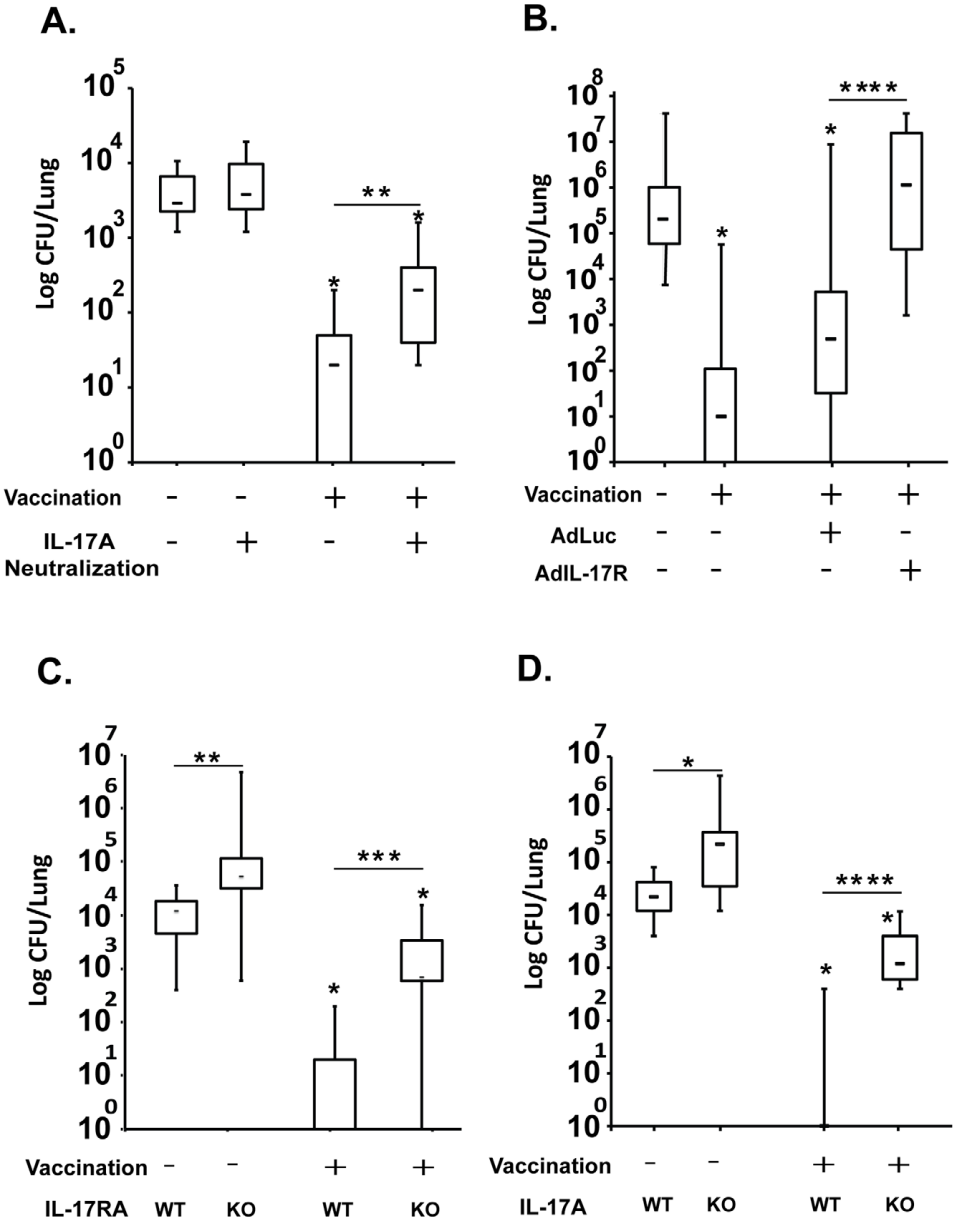
Non-redundant role of Tc17 cells in anti-fungal vaccine immunity. Mice were vaccinated with 10^5^ yeast of *Blastomyces* vaccine strain #55 subcutaneously (s.c.) and boosted once after 2 weeks. Two to three weeks later, mice were challenged intratracheally (i.t.) and lungs were harvested to enumerate CFUs. CD4^+^ T cells were depleted with GK1.5 mAb given i.v. weekly. **A.** Mice were given neutralizing anti-IL-17A mAb or rat IgG as control (100 µg each, i.v.) on days 0, 2 & 4 post-challenge. On day 6, lungs were analyzed for CFU. CFUs are from 8–14 mice/group. **B.** On day −3 and −1, mice were given 2–4×10^9^ pfu i.v. of recombinant adenovirus secreting mouse IL-17 receptor or control adenovirus expressing luciferase (AdLuc); a third dose was given on day 0 of challenge with virulent *Blastomyces* (i.t.). 10 days after challenge, lungs were harvested for CFU. CFUs are for 13–19 mice/group. **C.** IL-17RA^−/−^ and WT mice were infected i.t. with a lethal dose of WT *Blastomyces* yeast. 10 days later, lung CFU were enumerated. CFUs are from 10–14 mice/group from two independent experiments. **D.** IL-17A^−/−^ and WT mice were lethally challenged i.t. with WT *Blastomyce*s yeast. 13 days later, lung CFU were enumerated. CFUs are from 9–19 mice/group. In panels A–D, CFUs are shown in box and whisker plots. *, p<0.05; **, p<0.01; ***, p<0.001; and ****, p<0.0001.

The induction of a large number of Tc17 cells following fungal vaccination and their trafficking to the lung after experimental challenge led us to explore their functional role in vaccine resistance. We used three approaches. First, we neutralized IL-17A with monoclonal antibody (mAb) during the efferent phase of the immune response following pulmonary infection. Vaccinated CD4^+^ T-cell depleted mice that received anti-IL-17A mAb had >1 log more lung CFU after challenge than mice that got rat IgG control (**Fig. 2A**). In a second approach, we used recombinant adenovirus secreting soluble IL-17 receptor to neutralize circulating IL-17A in vaccinated mice depleted of CD4^+^ T cells. Mice that received the soluble IL-17 receptor had nearly 3 logs more lung CFU after challenge than mice that got the control adenovirus expressing luciferase (**Fig. 2B**). Thus, IL-17A is required during the efferent phase of vaccine immunity mice lacking CD4^+^ T-cells.

In a third approach, to study the roles of IL-17 and IL-17A/IL-17R signaling, we investigated vaccine resistance in IL-17 receptor A (IL17RA) knock out mice. Mouse T cells produce only IL-17A and IL-17F [37], [38], which both signal through IL-17RA. Thus, even if IL17RA^−/−^ mice develop vaccine-induced Tc17 and Tc1 cells, IL-17 could not signal via its receptor to mediate effector functions. Here, vaccinated IL-17RA^−/−^ mice depleted of CD4^+^ T cells had ≈2 logs more CFU in their lungs after challenge than did vaccinated wild-type (WT) mice (**Fig. 2C**). Thus, signaling through the IL-17A receptor is essential for controlling fungal pneumonia in vaccinated mice depleted of CD4^+^ T cells. Moreover, impaired IL-17A signaling reduced vaccine resistance, even in the presence of Tc1 cells.

### Tc17 cells are indispensable for vaccine-induced resistance but do not influence type I immunity

We have reported plasticity in anti-fungal vaccine immunity, with type 1 cytokines such as IFN-γ, TNF-α and GM-CSF contributing to resistance, but each having dispensable and compensatory roles for one another. We explored the obligate role of IL-17 using IL-17A^−/−^ mice depleted of CD4^+^ T cells. Vaccinated IL-17A^−/−^ mice had >3 logs more lung CFU after challenge vs. WT mice (**Fig. 2D**). Thus, Tc17 cells have an obligate, indispensable role in vaccine immunity in CD4^+^ T-cell deficient hosts.

It is possible that non-T cells are a source of IL-17 and resistance in vaccinated mice. We analyzed the lung cells of vaccinated CD4 depleted mice following pulmonary challenge. ∼82% (2.76/3.36%) of IL-17A^+^ cells were CD8^+^ T cells (Fig. S2). Thus, CD8^+^ T cells are the main cellular source of IL-17 in lung cells of vaccinated CD4 depleted mice.

**Figure 3:**
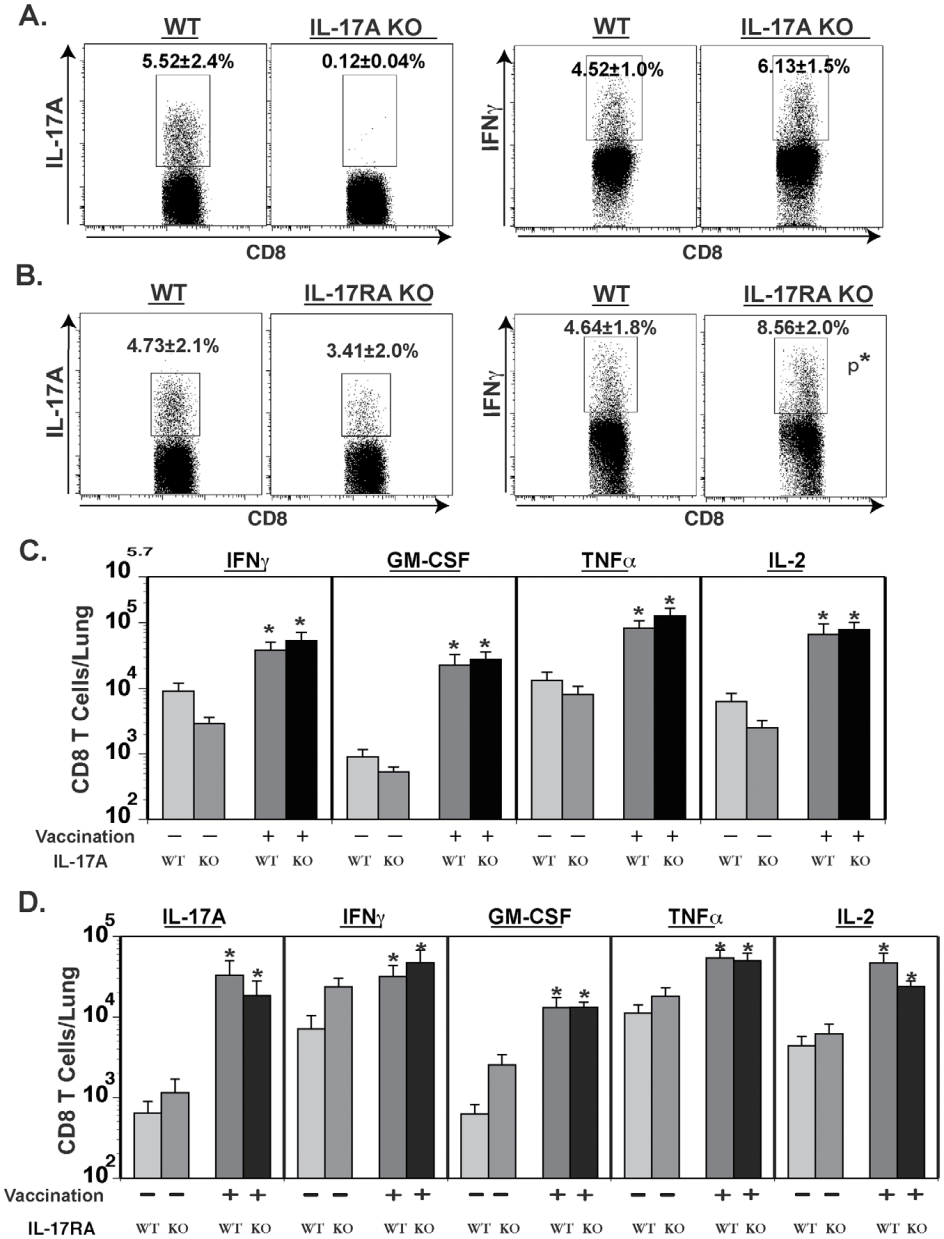
Cross-regulation of Tc17 and Tc1 cells during anti-fungal vaccine immunity. Mice were depleted of CD4^+^ T cells, vaccinated and challenged as described in Fig. 2. Percentage (**A & B**) and number (**C &D**) of cytokine-producing CD8^+^ T cells in WT, IL-17A^−/−^ (A & C) and IL-17RA^−/−^ (B & D) mice as measured by flow cytometry. Values are the mean ± SD of 4 mice/group. Results are representative of two independent experiments. *, p<0.05 vs. respective control.

Recent studies have shown cross-regulation of type 17 and type 1 immunity; the latter being augmented by the former during experimental infection with *Mycobacterium tuberculosis* or *Francicella tulerensis* [39], [40]. We studied cross-regulation of Tc1 by Tc17 cells in our vaccine model by analyzing CD8^+^ T cell responses in vaccinated IL-17A^−/−^ and IL-17RA^−/−^ mice after pulmonary challenge. The frequency of IFN-γ- and TNF-α-producing CD8^+^ T cells was not reduced in vaccinated IL-17A^−/−^ mice depleted of CD4^+^ T cells (**Fig. 3A** & data not shown). The frequency of IFN-γ producing cells was actually higher in vaccinated IL-17RA^−/−^ mice vs. WT controls (**Fig. 3B**). Overall, the numbers of Tc1 cells recruited to the lungs of vaccinated CD4^+^ T-cell depleted mice were maintained in IL-17A^−/−^ and IL-17RA^−/−^ mice vs. WT controls (**Fig. 3C & D**). Collectively, these data suggest that Tc17 cells are distinct and function independently of Tc1 cells in vaccine immunity to fungi. Tc17 cells neither reduced nor augmented type I immunity.

### Lack of IL-12 signaling does not alter Tc17 response or vaccine-induced CD8^+^ T cell immunity against lethal fungal pneumonia

**Figure 4:**
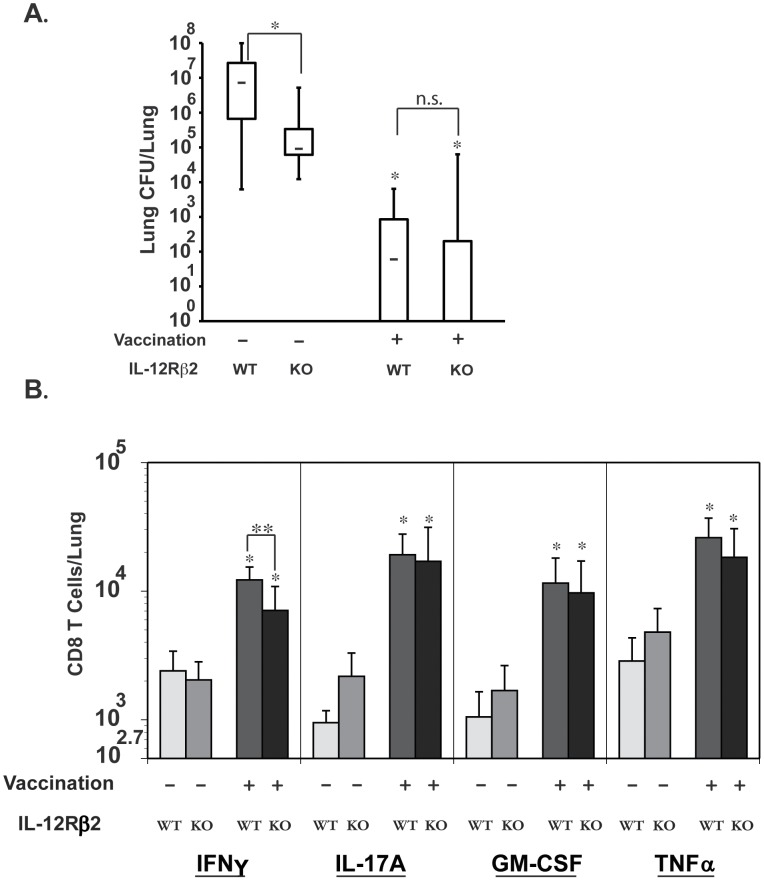
Role of IL-12 signaling on vaccine-induced Tc1 and Tc17 cells and resistance against fungal pneumonia. Mice were depleted of CD4^+^ T cells, vaccinated and challenged as described in Fig. 2. **A.** Three weeks after challenge, lung CFU were enumerated. CFUs are depicted in box and whisker plots for 10–17 mice/group. Data is representative of two independent experiments. **B.** On day 3–4 post-challenge, lungs were collected to enumerate cytokine producing CD8^+^ T cells by flow cytometry. Results are the mean ± SD of 4–8 mice/group from two independent experiments. *, p<0.05 vs. unvaccinated mice; and **, p<0.01.

IL-12 signaling polarizes the T cell response towards type 1 immunity and down modulates type 17 responses. Hence, lack of IL-12p35 enhances Th17 responses in *A. fumigatus* infection in mice [27]. We asked whether inhibiting type 1 responses have any impact on type 17 responses and vaccine-induced fungal immunity. We assayed resistance in vaccinated IL-12 receptor beta 2 (IL-12Rβ2) knock out mice depleted of CD4^+^ T cells. Surprisingly, loss of IL-12 signaling did not perturb vaccine immunity (**Fig. 4A**), since lung CFU values after challenge were similar in knockout and WT mice. Interestingly, abrogation of IL-12 signaling significantly enhanced resistance against pulmonary infection in unvaccinated knockout mice compared to WT controls (**Fig. 4A**).

We investigated how loss of IL-12 signaling influenced the recall responses of Tc1 and Tc17 cells to the lungs after pulmonary infection. Vaccinated IL-12Rβ2^−/−^ mice lacking CD4^+^ T cells had significantly less IFN-γ transcript and fewer numbers of IFN-γ producing CD8^+^ T cells in the lungs than WT controls (**Fig. 4B**; and data not shown). However, loss of IL-12 signaling did not affect lung transcript expression of IL-17A or IL-4, nor the recruited numbers of IL-17A, GM-CSF or TNFα –producing CD8^+^ T cells (**Fig. 4B**; data not shown). Collectively our data suggest that lack of IL-12 signaling in CD4^+^ T-cell deficient hosts impairs Tc1 (IFN-γ) responses, but this neither skews Tc17 responses nor alters vaccine resistance mediated by these cells against lethal pulmonary blastomycosis.

### Role of IL-17A signaling in neutrophils during Tc17 mediated anti-fungal vaccine immunity

**Figure 5:**
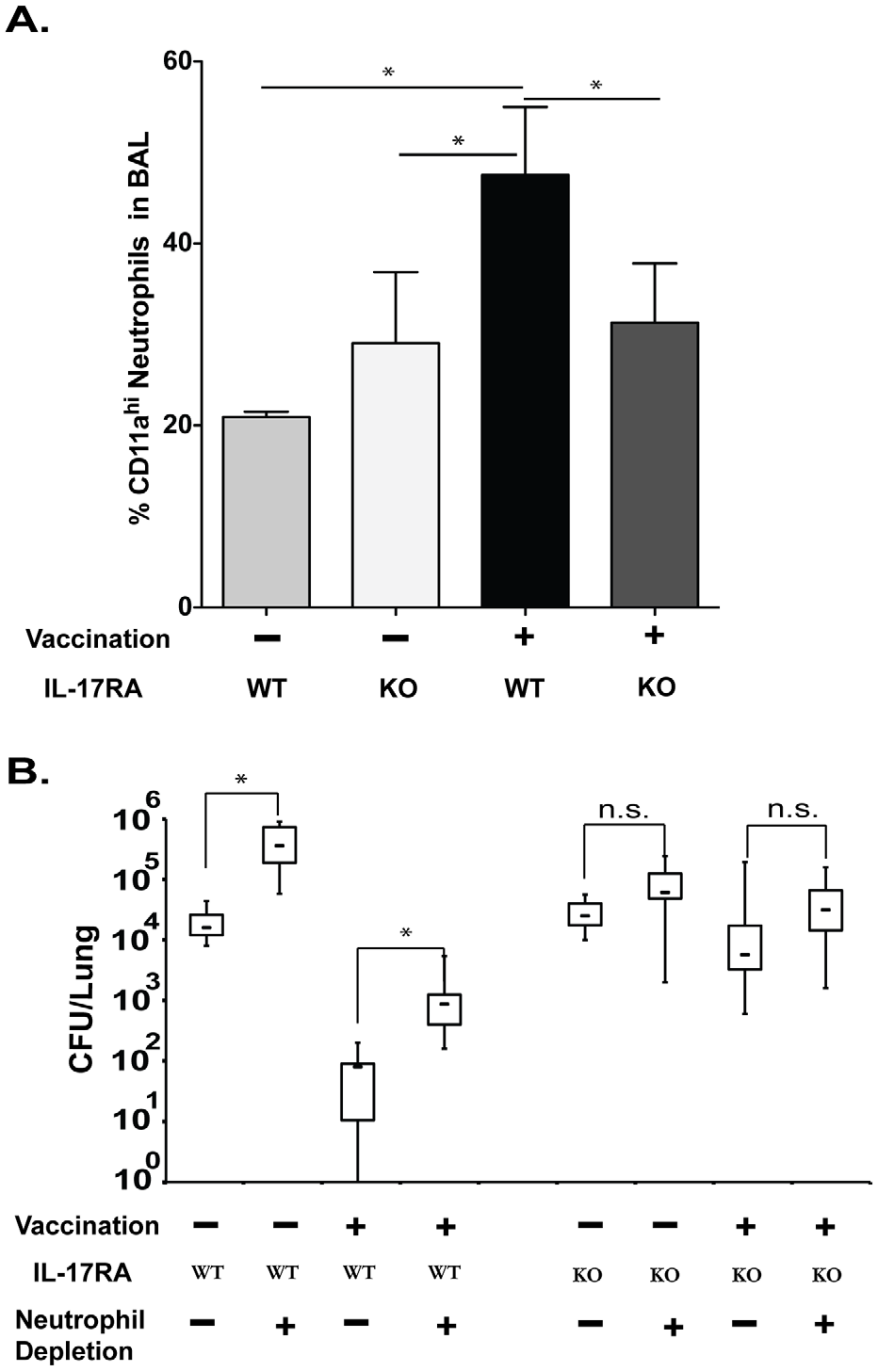
Role of IL-17A signaling in neutrophils during Tc17 mediated anti-fungal vaccine immunity. Mice were depleted of CD4^+^ T cells, vaccinated and challenged as described in Fig. 2. **A.** On day 4 post-challenge, BAL fluid was collected for enumeration of the percent Gr1^+^CD11a^hi^ (LFA-1^+^) neutrophils by flow cytometry. Values are mean ± SD of 4–5 mice/group. Data is representative of two independent experiments. **B.** Lung CFUs at day 6 post-challenge. 100 µg of mAb 1A8 was used at days 0, 2 and 4 post-challenge to deplete neutrophils; ≥99% of the cells were depleted as measured by flow cytometry (data not shown). CFUs are in box and whisker plots for 6–18 mice/group. Data is representative of two independent experiments. *, p<0.05 for comparison indicated.

Th17 immunity promotes infiltration and activation of neutrophils to sites of infection. We studied the mode of action of Tc17 cells and tested whether neutrophils promote Tc17 vaccine immunity against *Blastomyces*. To address this, we used IL-17RA^−/−^ mice in which IL-17A signaling is abolished on responding cells, including neutrophils, and analyzed LFA-1^+^ neutrophils in the BAL fluid in vaccinated CD4^+^ T-cell depleted mice after challenge. The frequency of LFA-1^+^ neutrophils recruited to the lungs was significantly higher in vaccinated WT mice compared to vaccinated IL-17RA^−/−^ mice or unvaccinated controls (**Fig. 5A**). To functionally test the role of neutrophils in vaccine resistance, we used monoclonal antibody to selectively deplete Ly6G^+^ neutrophils during the effector phase or recall response to pulmonary infection. Vaccinated WT mice that were depleted of neutrophils had 37-fold more lung CFU than WT controls given rat IgG (**Fig. 5B**). Unvaccinated WT mice depleted of neutrophils also had higher lung CFU values than control littermates that got rat IgG – approximately 17-fold (**Fig. 5B**). Thus, a significant component of the resistance mediated by neutrophils was attributable to vaccination in WT mice. In contrast, in IL-17RA^−/−^ mice, depletion of neutrophils had a negligible effect on lung CFUs in vaccinated mice (and also unvaccinated mice). Thus, IL-17 signaling is essential for the infiltration and activation of neutrophils in vaccinated mice depleted of CD4^+^ T cells. These data suggest that Tc17 cells employ neutrophils as a mode of action in mediating vaccine immunity against fungal infection.

We considered that neutrophils recruited in response to IL-17 in vaccinated mice might be responsible for exuberant immunity and excessive damage. To address this issue, we analyzed lung histopathology (**Fig. S3**). Vaccinated mice deficient in IL-17 signaling had extensive lung inflammation after infection, whereas vaccinated wild-type mice had the least inflamed lungs. IL-17A^−/−^ mice gave similar results (data not shown). Thus, IL-17 was associated with better control of the infection and less inflamed lungs rather than more inflammation in this model.

### Role of Dectin-1, IL-1 and IL-6 in eliciting protective Tc17 cells

**Figure 6:**
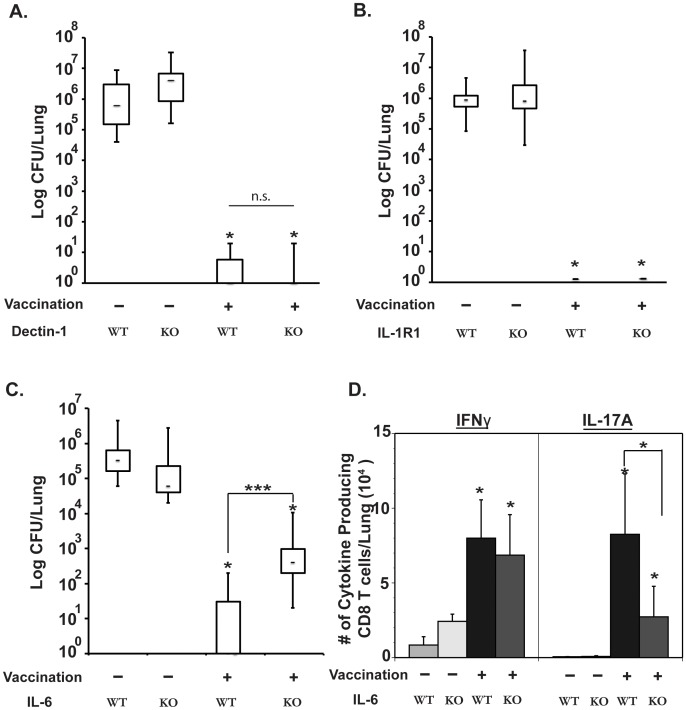
Roles of Dectin-1, IL-1R1 and IL-6 in the induction of Tc17 cells and anti-fungal vaccine immunity. Mice were depleted of CD4^+^ T cells, vaccinated and challenged as described in Fig. 2. **A, B and C**. Lung CFU values (in box and whisker plots). Three weeks post-challenge in Dectin-1^−/−^ and WT controls (N = 7–10/group) (**A**); 17 days post-challenge in IL-1R1^−/−^ mice and WT controls (N = 10–13/group) (**B**); and day11 post-challenge in IL-6^−/−^ mice and WT controls (N = 5–11) (**C**). **D.** Number of cytokine-producing cells recruited to the lungs 4 days after challenge of IL-6−/− and WT control mice, as measured by flow cytometry. *, p<0.01 vs. unvaccinated controls unless otherwise indicated.

We investigated elements that regulate the differentiation of Tc17 cells that mediate anti-fungal vaccine immunity. In many fungal infection models, Dectin-1 and IL-1 are instrumental for the induction of Th17 cells [2]. In a recent study [41], IL-1 was found to be indispensable during differentiation of Th17 cells, whereas IL-6 was required at some but not all compartments. We looked into these pathways since little is known about the differentiation of anti-fungal Tc17 cells. The differentiation of Tc17 cells was unimpaired in vaccinated Dectin-1^−/−^ and IL1-R1^−/−^ mice depleted of CD4^+^ T cells as compared to vaccinated WT littermates. The numbers of Tc17 cells in the lung during recall responses were comparable among these groups (**Fig. S4A & B, and S5A**). Similarly, vaccinated Dectin-1^−/−^ and IL-1R1^−/−^ mice depleted of CD4^+^ T cells were as resistant to pulmonary infection as vaccinated WT littermates. Each group had lung CFU values nearly 6 logs lower than unvaccinated controls (**Fig. 6A & B**). In contrast, vaccinated IL-6^−/−^ mice depleted of CD4^+^ T cells harbored ≈3 logs more lung CFU after challenge compared to vaccinated WT mice, even though vaccinated IL-6^−/−^ mice were significantly more resistant than unvaccinated WT littermates (**Fig. 6C**). The numbers of Tc17 cells detected in the dLNs and spleen of vaccinated mice and in their lungs after challenge were significantly lower in IL-6^−/−^ mice as compared to WT mice (**Fig. 6D** **& S5B**). Thus, IL-6 was essential for the induction of Tc17 cells and anti-fungal vaccine immunity, whereas both Dectin-1 and IL-1R1, were each dispensable for the induction of Tc17 cells and vaccine-induced resistance in CD4^+^ T-cell depleted mice.

### CCR6 not CXCR3 is required for recruitment of Tc17 cells into the lung

**Figure 7:**
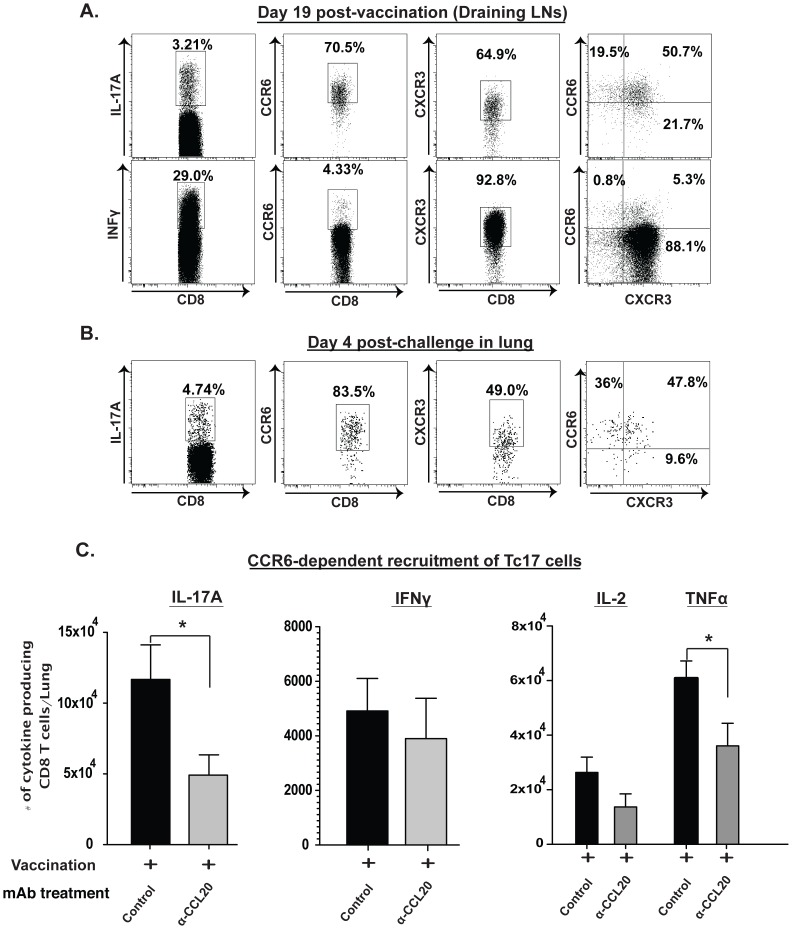
CCR6 mediated recruitment of Tc17 cells into the lung after challenge. Mice were depleted of CD4^+^ T cells and vaccinated as described in Fig. 2. **A.** Dot plots show the frequency of CCR6^+^ or CXCR3^+^ cells (middle and right panels) gated on the respective cytokine producing CD8^+^ T cells (left panel) in the dLNs after vaccination. **B.** Dot plots show the frequency of CCR6^+^ or CXCR3^+^ Tc17 cells (middle and right panels, respectively) in the lung 4 days after pulmonary challenge. **C.** The number of cytokine producing CD8^+^ T cells in the lung 5 days after pulmonary challenge as measured by flow cytometry. For neutralization of CCL20, vaccinated mice were given ∼90 µg of α-mouse CCL20 mAb or control antibody i.v. on days 0, 2 and 4 post-challenge. Values are mean ± SD of 5–6 mice/group. *, p<0.05.

We have found that the chemokine receptor, CXCR3 mediates the recruitment of vaccine-induced anti-fungal IFN-γ producing CD8^+^ T cells [36]. The chemokine receptor CCR6 is thought to be preferentially associated with Th17 cells [42], but its expression and function on Tc17 cells has not been studied. We examined the expression of chemokine receptors on vaccine induced Tc17 cells and their function in recruiting these cells into the lung. Following vaccination and analysis of cells in dLNs, nearly 70% of Tc17 cells expressed either CXCR3 or CCR6, whereas IFN-γ producing CD8^+^ T cells preferentially expressed CXCR3 (**Fig. 7A**). Among Tc17 cells that were recruited to the lungs of vaccinated mice after infection, the expression of CCR6 (84%) was much higher than that of CXCR3 (49%) (**Fig. 7B**). In view of co-expression of these receptors on a substantial number of Tc17 cells, we tested their functional role in recruitment to the lung. Antibody blocking of the CXCR3 receptor during recall did not affect recruitment of Tc17 cells (data not shown), although it did affect the recall of IFN-γ producing CD8^+^ T cells [36]. To test the role of CCR6 on Tc17 cells, we neutralized its chemokine ligand CCL20 during the effector phase (or recall response) after infection. Recruitment of Tc17 cells was sharply reduced in mice that received α-CCL20 as compared to control rat IgG (**Fig. 7C**). Treatment with α-CCL20 did not perturb the infiltration of IFN-γ^+^ or IL-2^+^ CD8^+^ T cells. Interestingly, TNF-α-producing CD8^+^ T cells were also reduced in mice treated with α-CCL20, but the reduction in double positive cells (TNFα^+^IL-17^+^) could not account for the reduction of total TNFα^+^ CD8^+^ T cells (**Fig. 7C** & data not shown). Thus, CCR6 mediates the recruitment of Tc17 cells to the lung during recall after pulmonary fungal infection.

### Profile of vaccine induced Tc17 cells predicts persistence and stem cell likeness

**Figure 8:**
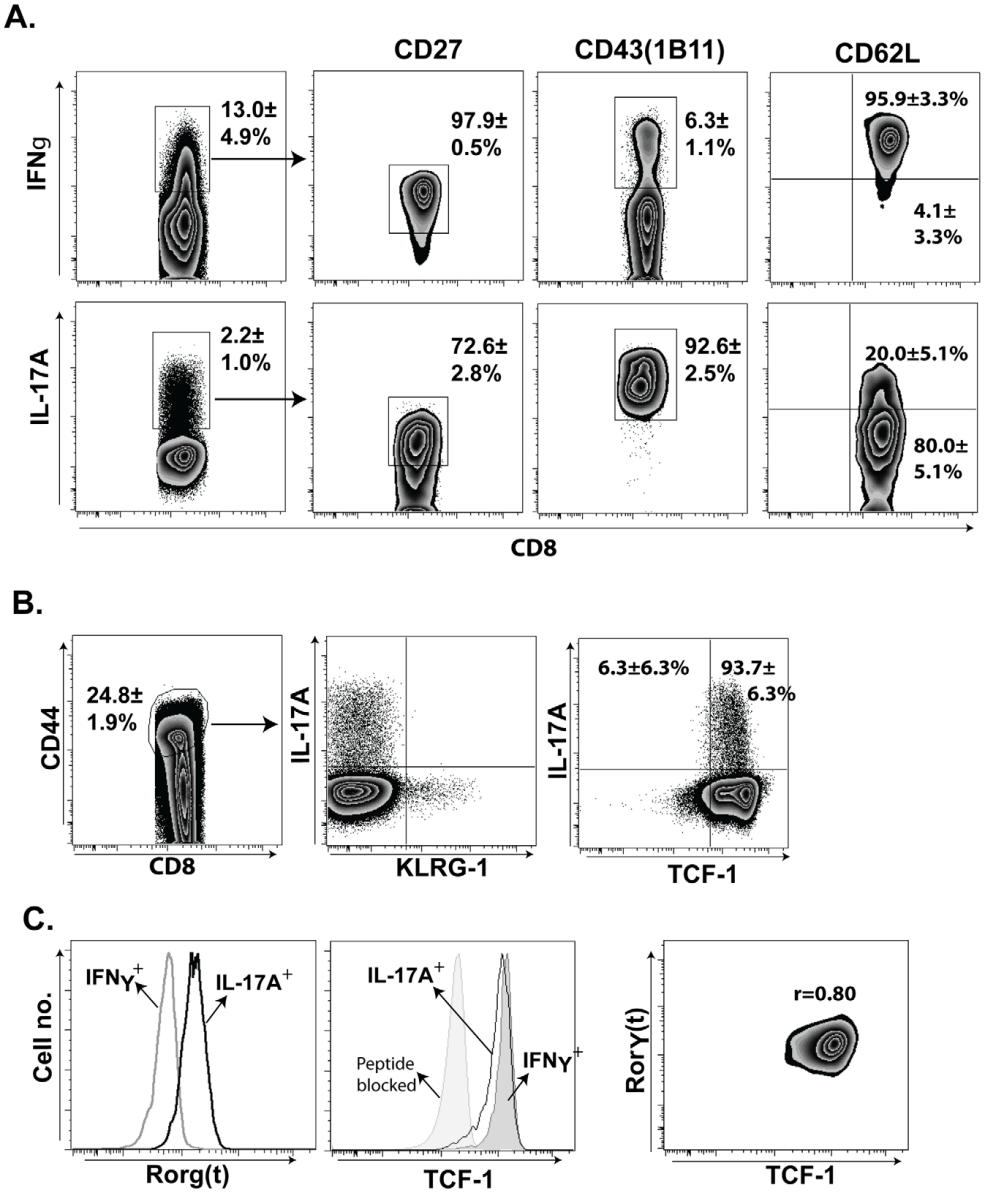
Phenotypic attributes of anti-fungal effector Tc17 cells. Mice were depleted of CD4^+^ T cells and vaccinated once as described in Fig. 2. On day 19, dLNs cells were harvested and stimulated *ex vivo* with anti-CD3 and -CD28 in the presence of Golgi stop and TAPI-2 at 37°C for 5 hrs. Following incubation, cells were surface-stained before staining for intracellular cytokine or transcription factor using phospho-staining kit as in Methods. **A.** Zebra plots showing percent CD27, CD43 & CD62L of IFN-γ^+^ and IL-17A^+^ CD8^+^ T cells. Values are mean ± SD of 4–5 mice/group. **B.** Zebra plots show frequency of IL-17^+^, KLRG-1^+^ and TCF-1^+^ among CD8^+^CD44^+^ cells. Values are mean ± SD of 4–5 mice/group. **C.** Left two panels show mean fluorescence (MFI) of RORγt and TCF-1 on IFN-γ^+^ and IL-17A^+^ CD8^+^ T cells. Right panel shows correlation of RORγt and TCF-1 expression on IL-17A^+^ CD8^+^ T cells. r = correlation coefficient derived from MFIs. Data are representative of two-independent experiments.

Th17 cells reportedly survive poorly after *Listeria* infection due to their inability to maintain CD27 expression [35]. However, we recently found that Tc17 and Tc1 cells can be recalled into the lungs even after 6 months of rest in vaccinated CD4^+^ T-cell deficient hosts, suggesting that they can persist [36]. Consistent with our findings, a recent study found that, irrespective of CD27 expression, Th17 cells can exhibit stem-cell like features and survive for long periods in a manner that correlates with expression of the stem-cell like transcription factor TCF-.1 [34]. We therefore assessed the surface and transcriptional profile of Tc17 cells, while also contrasting it with Tc1 cells. After fungal vaccination, Tc17 cells are mainly CD43^hi^ (∼93%), ∼70% are CD27^hi^ and most are also CD62L^lo^ (∼80%) (**Fig. 8A**). In sharp contrast, IFN-γ^+^ Tc1 cells are chiefly CD43^lo^ (∼7%), CD27^hi^ (∼98%), and CD62L^hi^. Tc17 cells expressed the lineage-specific transcription factor RORγt and showed a phenotypic profile of memory precursors i.e. KLRG1^lo^ and TCF-1^hi^ (**Fig. 8B & 8C** **, and** **S6**). Expression of the T17 prototypic transcription factor RORγt correlated strongly with the expression of TCF-1 (**Fig. 8C**; r = 0.80). These data suggest that fungal vaccine-induced Tc17 cells are full effector cells, but portray a stem cell-like phenotype that has been observed in memory T-cell precursors [34].

## Discussion

Our study shows that Tc17 cells can be induced irrespective of CD4^+^ T cell help upon fungal vaccination. Like Th17 cells, Tc17 cells are non-redundant in mediating fungal resistance [13]. We used four different approaches to eliminate IL-17 or its activity in vaccinated CD4 depleted mice: neutralizing antibody against IL-17A or soluble IL-17A receptor during recall responses and IL-17A and IL-17RA knockout mice. All indicated an unequivocal and distinct role for IL-17A in vaccine resistance in CD4 depleted mice. Our data do not formally prove that Tc17 cells mediate vaccine resistance in this model, since it is impossible to selectively deplete Tc17 cells while leaving behind other Il-17 producing lymphoid cells. Yet, the fact that nearly 85% of the IL-17A producing cells in the lung of vaccinated CD4-depleted mice are CD8^+^ T cells, and that depletion of CD8^+^ T cells in this model eliminates vaccine resistance [19] supports our conclusion that Tc17 cells are non-redundant and indispensable for vaccine resistance in this model.

In a *M. tuberculosis* infection model, Th17 cells mediated resistance by recruiting anti-bacterial Th1 cells into the lungs. However, our studies with IL-17A and IL-17A receptor knockout mice showed that the recruitment of functional anti-fungal Tc1 cells was not impaired in this setting. Yet, the fungal clearance was dramatically blunted in the absence of Tc17 cells, indicating the unique and indispensable role of Tc17 cells in anti-fungal resistance. We did not see significant numbers of dual type I and type 17 cytokine-producing T cells during recall responses on pulmonary infection (data not shown). Our observations are in line with the recent study in a *Klebsiella* infection model where Th17 cells conferred distinct anti-microbial function independent of Th1 immunity [43].

Our previous work showed that Tc1 immunity in CD4^+^ T cell deficient hosts was pivotal for vaccine-induced resistance against blastomycosis and histoplasmosis [19]. The type 1 cytokines IFN-γ, TNFα and GM-CSF played critical and overlapping roles in the clearance of pulmonary fungal infection. Our current study adds the additional cytokine IL-17A produced from CD8^+^ T cells. In fact, when IL-12 signaling is nullified, vaccine-induced resistance was intact even though there were reduced numbers of IFN-γ producing cells. Unlike the situation of CD4^+^ T-cell immunity in *A. fumigatus* infection [27], we found that a lack of IL-12 signaling did not abate or enhance Tc17 cells, indicating minimal cross-talk between the two pathways - Tc1 and Tc17 - in our model of anti-fungal vaccine immunity. Moreover, Tc17 cells mediate anti-fungal vaccine immunity independently of Tc1 cells. Tc17 immunity did not influence Tc1 immunity, and vice versa, suggesting the unique, indispensable role of Tc17 cells for fungal resistance in the absence of CD4^+^ T cells.

Although the anti-microbial actions of IL-17A are under investigation, several studies have reported its role in recruiting and activating neutrophils [44]. Here, we showed that Tc17 cells likely play a critical role in activating neutrophils. Depletion of neutrophils during the efferent/recall response reduced fungal clearance and this effect was dependent on IL-17A signaling since there was no effect of depletion on fungal resistance in IL-17RA knockout mice.

Dectin-1 promotes anti-fungal defense by inducing Th17 cells in the setting of infection with C. *albicans*, A. *fumigatus*, and P. *carinii* [11], [45], [46]. Dectin-1 can promote the induction of Th17 cells by inhibiting Th1 differentiation [27]. Our work shows that Dectin-1 is dispensable for eliciting Tc17 cells and promoting vaccine resistance to *Blastomyces* in CD4^+^ T-cell deficient hosts. Similarly, IL-1R1, which has been shown to be essential for the induction of Th17 cells in different microbial or non-microbial models, was found here to be dispensable for inducing Tc17 and controlling fungal infection in vaccinated CD4^+^ T-cell deficient hosts. Conversely, we found that a classical inducer of the Th17 lineage, IL-6, was essential in induction of Tc17 cells. It is noteworthy that Dectin-1 activation leads to production of IL-1, which in turn can induce and amplify production of IL-6 [28], [47], [48]. Our work indicated that even though IL-6 is required for induction of Tc17, its production was independent of Dectin-1 or IL-1 receptor signaling suggesting the involvement of different pathogen-recognition receptor(s). Further studies may reveal distinct or overlapping functions of different PRRs in the induction of IL-6.

Th17 cells express the chemokine receptor CCR6, which promotes their trafficking to mucosal surfaces [42], [49]. Although T cells may co-express different chemokine receptors, CCR6 is distinctly expressed on Th17 cells [42], [50] due to the upstream influence of the transcription factor RORγt. Conversely, CXCR3 regulates the trafficking of type 1 cytokine producing cells [51]. Surprisingly, we observed that Tc17 cells expressed both CXCR3 and CCR6 in the dLNs and after recall to the lungs [52]. We did not see dual expression of IFN-γ and IL-17A in these Tc17 cells. Nevertheless, CCL20 neutralization and CXCR3 blocking experiments showed that CCR6 but not CXCR3 is critical in mobilizing Tc17 cells from lymphoid organs to the lungs for vaccine resistance against fungal pneumonia. This feature of dual chemokine receptor expression marks an additional layer of Tc17 differentiation following fungal vaccination. Dual expression may be due to Tc17 plasticity during the initial differentiation stages after vaccination or a stochastic phenomenon of these T cells.

CD4^+^ T cell help was dispensable for the induction of Tc17 cells and their recall to the lung. This finding may have implications for designing anti-fungal vaccines targeted to immune-compromised patients. Although CD8^+^ memory immunity against bacteria and viruses wanes quickly in the absence of CD4^+^ T cell help [53], we have found that vaccine induced anti-fungal CD8^+^ T cells do acquire long-term memory and can be maintained in the absence of CD4^+^ T cell help [36]. While antigen-specific Th17 cells have been thought to be short lived [35], and exhibit plasticity toward a Th1 phenotype [54], a recent study found that Th17 cells evince stem-cell likeness and multi-potency in cytokine production [34]. Such Th17 cells displayed a unique transcriptional signature allowing them, despite plasticity and conversion toward a Th1 phenotype, to retain their ability to produce type 17 cytokines and reject tumors more effectively than distinct Th1 lineage cells. Here, we found that Tc17 cells were indispensible in vaccine resistance to lethal fungal infection in mice rested for a month after vaccination. Indeed, in a recent study [36], we found that Tc17 cells persisted 6 months after vaccination in CD4^+^ deficient hosts and could be recalled to the lungs after challenge (though we did not test their role in resistance). In the current study, we extend those findings by demonstrating that the phenotype and transcription factor profile of these Tc17 cells after vaccination, even in the absence of CD4^+^ T cells, show features that portend long-term persistence and stem-cell likeness.

Herein, vaccine induced effector Tc17 cells were distinct in their surface phenotype as compared to effector Tc1 cells. They were chiefly CD43^hi^ and CD62L^lo^. The role of CD43 on Tc17 cells has not been studied, but other work has shown that it promotes expansion, contraction and tissue trafficking [55], [56]. CD43 expression on CD8^+^ T cells has been associated with enhanced clonal burst size during the expansion phase and increased tissue trafficking, suggesting its positive role during the early phase of an immune response [47], [57]. During later phases, it potentiates apoptosis of CD8^+^ T cells, down-regulating immune responses and immunopathology [55]. Thus, CD43 on Tc17 cells after fungal vaccination may promote tissue trafficking to the lungs or tone down immunopathology associated with IL-17A production. Although we have shown that Tc17 cells are maintained in vaccinated mice [36], we did not assess CD43 expression on those cells. CD43 may be down regulated on these persistent cells, or alternatively, CD43 function may differ on persistent anti-fungal Tc17 cells versus anti-viral Tc1 cells [58].

Vaccine induced Tc17 were fully differentiated effectors, but not terminally differentiated as indicated by their phenotype: CD44^hi^, CD43^hi^, CD62L^lo^, KLRG-1^lo^ and TCF-1^hi^. Only ∼20% of Tc17 cells were CD62L^hi^ denoting them as central memory cells. The remaining 80% of Tc17 cells were CD62L^lo^ yet also KLRG-1^lo^ and TCF-1^hi^ suggesting they have the propensity to become memory cells [34], [59], [60]. Although, we did not look at the long-term fate of these cells, an important question is whether they remain as effector memory cells and traffic to peripheral tissues or convert into a central memory phenotype during the memory phase.

TCF-1 can repress T17 lineage development by directly binding the IL-17A locus [61], [62]. Here, we showed that RORγt expression in Tc17 cells was strongly correlated with the expression of TCF-1. It is possible that the quality or strength of the fungal vaccine signal for Tc17 cell differentiation was sufficient to convert them to fully but not terminally differentiated effector cells without down-regulating TCF-1, and to overcome TCF-1 inhibition of IL-17A expression. The quality and quantity of TCR signals regulating TCF-1 require further investigation. Understanding the features that foster the long-term survival and function of Tc17 cells are important for developing vaccine strategies that prevent fatal fungal disease in immune-compromised patients, where Tc17 cells are known to exert and indispensable role in protective immunity.

## Methods

### Ethics statement

All animal procedures were performed in accordance with the recommendations in the Guide for the Care and Use of Laboratory Animals of the National Institutes of Health. Care was taken to minimize animal suffering. The work was done with the approval of the IACUC of the University of Wisconsin-Madison.

### Mouse strains

Wild type C57BL/6 mice were obtained from the National Cancer Institute. Breeder pairs of IL17ra^−/−^ and IL17a^−/−^ mice were provided by Amgen and Jay Kolls (University of Pittsburgh, Pittsburgh, Pennsylvania, USA), respectively. Breeder pairs of *Il12rb2^−/−^* B6.129S1-*Il12r*β*2^tm1Jm^*/J (stock 3248); *Il1r1^−/−^* B6.129S7-*Il1r1^tm1Imx^*/J (stock 003245); B6.129S2-Il6^tm1Kopf/^J (stock 002650); C57BL/6-Tg (TcrαTcrβ) ^1100Mjb/J^ (stock 003831) mice (referred to as OT-I mice in this paper); B6.129S2-Cd4^tm1Mak^/J (stock 002663); and T lymphocyte–specific Thy 1.1 allele-carrying congenic B6 strain B6.PL-Thy1^a^/Cy (stock 000406) were purchased from Jackson Laboratories. OT-I Tg mice were bred on Thy1.1 congenic mice to generate Thy1.1^+^ OT-I Tg mice. Dectin-1^−/−^ mice were a kind gift from Dr. Gordon Brown (University of Aberdeen, Scotland). All mice were 7–8 weeks of age at the time of experiments. Mice were housed and cared for according to strict guidelines of the University of Wisconsin Animal Care Committee, who approved all aspects of this work.

### Fungi

The wild-type virulent strain of *Blastomyces dermatitidis* is American Type Culture Collection (ATCC) 26199 and was obtained from ATCC. The isogenic, attenuated mutant lacking BAD1, designated strain #55, was used for vaccination. Isolates of *B. dermatitidis* were maintained as yeast on Middlebrook 7H10 agar with oleic acid-albumin complex (Sigma-Aldrich) at 39°C. For OT-I responses, recombinant strain #55 carrying the OT-I epitope, ovalbumin SIINFEKL, was used for vaccination and was maintained as isolates of strain #55 as described [36]. In some of the experiments, *H. capsulatum* strain G21B was used, which was maintained on *Histoplama* Macrophage Medium (HMM) plates.

### Adoptive transfer of OT-I cells

OT-I cells from lymph nodes and spleens of OT-I Tg mice were purified using CD8^+^ T-cell negative enrichment magnetic beads kit (BD Biosciences). A total of 1×10^6^ naïve OT-I cells were adoptively transferred to naïve congenic mice by the intravenous (i.v.) route.

### Vaccination and experimental infection

Mice were vaccinated with 10^5^–10^6^ yeast of attenuated *B. dermatitidis* (#55 strain) by the subcutaneous (s.c.) route at each of two sites, dorsally and at the base of the tail. For challenge studies, mice were infected intratracheally (i.t.) with ∼2×10^3^ yeast of the isogenic wild-type strain of *B. dermatitidis,* ATCC 26199. To assess yeast burden, lungs were homogenized before plating on brain heart infusion (BHI; Difco) agar. For OT-I T cell studies, mice were vaccinated with 10^6^–10^7^yeast of recombinant vaccine strain #55 yeast expressing OVA SIINFEKL.

### CD4^+^ T-cell depletion

All experimental mice, unless stated, were depleted of CD4^+^ T-cells with monoclonal antibody GK1.5 (Biovest International Inc./NCCC, MN) using a weekly dose of 100 µg given by the i.v. route. The efficiency of depletion of CD4^+^ T cells was ≥99% as measured by flow cytometry [36]; ≈5% of depleted cells are Thy1.2 negative.

### Intracellular cytokine staining and FACS analysis of CD8^+^ T cells

Lymphocytes from the draining lymph nodes (dLNs), spleen and lung were obtained after homogenization and lysis of RBCs. Cells were re-stimulated with anti-CD3 (clone 145-2C11; 0.1 µg/ml) and anti-CD28 (clone 37.51; 1 µg/ml) in the presence of Golgi-Stop for 5 hrs at 37°C. Following incubation, cells were washed and surface stained with anti-Thy1.1, anti-CD8α and anti-CD44 antibodies. In some experiments, cells were also stained with anti-CD27, anti-CD43 (1B11), anti-KLRG-1 and anti-CD62L antibodies. Cells were washed to remove unbound antibodies and were fixed and permeabilized using a Cytofix/Cytoperm kit. Cells were then stained for intracellular cytokines using anti-IFN-γ, anti-IL-17A, anti-TNF-α and anti-IL-2 antibodies. For staining of chemokine receptors, anti-CCR6 and anti-CXCR3 antibodies were added during surface staining. All antibodies and staining reagents were obtained from BD Biosciences except for the anti-CXCR3 antibody, which was obtained from Biolegend. Cells were analyzed by flow cytometry.

### Analysis of fungal-antigen specific CD8 T cell responses

∼2×10^6^ bone marrow derived dendritic cells (BMDCs) were incubated with ∼2×10^6^ CFU of heat-killed vaccine yeast overnight at 37°C. On the following day, ∼1×10^6^ dLNs cells or lung cells were added to the culture along with Golgi stop and incubated for an additional 5 hrs. Cells were subjected to surface and intracellular staining before flow cytometric analysis.

### *In vivo* neutralization of IL-17 and CCL20

To neutralize soluble IL-17A, mice were given 100 µg of anti-IL-17A mAb by the i.v. route on days 0, 2 and 4 after pulmonary infection as described [13], which efficiently neutralizes IL-17 [63]. To neutralize IL-17 with the soluble receptor, mice were infected with recombinant adenovirus expressing soluble IL-17RA at a dose of 2–4×10^9^ pfu on days −3 and −1 (i.v. route) and on day 0 (i.t. route at the time of *Blastomyces* infection). As a control, we used adenovirus AdLuc expressing luciferase (provided by Jay Kolls and propagated by the Vector Core lab at the University of Michigan, Ann Arbor, MI). For neutralization of chemokine CCL20, mice were given ∼90 µg of α-mouse CCL20 mAb (R&D Systems) by the i.v. route.

### FACS analysis of neutrophil population in BAL fluid

A total of 10 ml of BAL fluid was harvested by repetitive instillation of 1 ml cold PBS plus 0.05% EDTA via the i.t. route. Cells were washed and re-suspended in FACS buffer and surface stained with Violet Live/Dead stain (Molecular Probes/Invitrogen), Ly6G-APC (clone 1A8), LFA1-PE, CD11b-PECy7, Ly6G-APC, and 7/4-biotin with streptavidin PerCPCy5.5 (BD Bioscience). Cells were kept on ice throughout the procedure. Cells were fixed and analyzed by flow cytometry.

### Neutrophil depletion

Vaccinated and unvaccinated mice were injected by the i.v. route with 100 µg of anti-mouse Ly6G mAb (clone 1A8; BioXCell) on days 0, 2, and 4. Depletion (99% efficient) was confirmed by FACS analysis of cells in the lung homogenate. As a control, mice were given similar amounts of rat IgG antibody (Sigma-Aldrich).

### Flow cytometric analysis of TCF-1 by phospho-protein staining

Cells from dLNs were harvested, stimulated with anti-CD3 and anti-CD28 at 37°C for 5 hrs in the presence of Golgi-stop. In some experiments, TAPI-2 was added to inhibit shedding of CD62L [64]. Following incubation, cells were surface stained in FACS buffer. Cells were then fixed/permeabilized using Phosflow Lyse/Fix buffer and Phosflow Perm/Wash buffer I (BD Biosciences), blocked with buffer containing normal goat sera and stained with rabbit anti-mouse TCF-1, anti-IL-17A, anti-IFNγ and RORγt. As a control for antibody staining, we blocked the staining antibody with the peptide used to generate TCF-1 antibody (Cell Signaling Technology). After washing, cells were stained with goat anti-rabbit secondary antibody. Cells were washed and analyzed by flow cytometry.

### Statistical analysis

Statistical significance of differences in fungal lung CFU was measured by the non-parametric Mann-Whitney test. All other statistical analysis was performed using a two-tailed unpaired Student t test. A two-tailed P value of ≤0.05 was considered statistically significant.

## Supporting Information

Figure S1 **Tc17 cells are recalled in the lung in the absence of CD4^+^ T cells upon** ***Histoplasma capsulatum*** **infection.** Mice were vaccinated s.c. with ∼10^6^ cfu of H. *capsulatum* yeast. A weekly dose of 100 µg GK1.5 mAb was used to deplete CD4^+^ T cells. After 8–9 wks, mice were challenged intratracheally with sublethal dose of 2×10^5^ cfu and 4 days later the lungs were harvested to analyze cytokine producing CD8^+^ T cells by flow cytometry. **A.** Dot plot shows percent CD8^+^ T cells expressing IL-17A in CD4^+^ T-cell sufficient and depleted mice. **B.** Total number of cytokine-producing CD8^+^ T cells/lung in CD4^+^ T-cell sufficient and depleted groups. Values are mean ± SD of 3–5 mice/group. *, p<0.05.
(TIF)

Figure S2 **IL-17A producing cells in the lung of vaccinated mice after infection.** Mice were depleted of CD4+ T cells, vaccinated and intratracheally infected as described in Fig. 1 and 2. Lung cells were harvested, re-stimulated and assessed for IL-17A producing cells by flow cytometry. The numbers in the plot indicate the percent of cells among lymphocyte-gated total lung cells.
(TIF)

Figure S3 **Pulmonary inflammation in vaccinated mice in the absence of IL-17A signaling following infection.** Mice were depleted of CD4+ T cells, vaccinated and intratracheally infected as described in Fig. 1 and 2. Lung tissues were collected and stored in 10% neutral buffered formalin. Lung tissue sections were taken and stained with H&E for histopathology studies. Upper panels images are at 40× magnification; lower panels are at 200×. Vaccinated wild-type mice have mostly a peribronchiolar pattern of inflammation (asterisks), while the knockout mice exhibit mostly a perivascular pattern (arrows). Nodular bronchocentric foci of inflammation with bronchiolar exudate are present in the knockout mice (B), but these foci of inflammation are rare in wild type mice (A). The peribronchiolar and perivascular infiltrates in both wild-type (C) and knock out (D) mice are composed of lymphocytes, plasma cells, few intact neutrophils, and rare histiocytes. In contrast, the bronchiolar exudate (asterisks) and peribronchiolar nodular infiltrates are primarily histocytic with fewer neutrophils and lymphocytes (D).
(TIF)

Figure S4 **Dispensability of Dectin-1 and IL-1R1 signaling for vaccine-induced Tc17 cells recruited to the lung.** Groups of wild-type and Dectin-1^−/−^ mice were depleted of CD4^+^ T-cells and vaccinated as described in Fig. 2. Two weeks after the boost, mice were challenged intratracheally with 2×10^3^ cfu of wild-type yeast. Four days later, mice were sacrificed; lungs were harvested and analyzed for intracellular cytokine staining by flow cytometry. Total number of cytokine producing CD8^+^ T cells in Dectin-1^−/−^ (**A**) and IL-1R1^−/−^ (**B**) and wild-type mice. Values are mean ± SD of 5–6 mice/group.
(TIF)

Figure S5 **Non-redundant role of IL-6, but not Dectin-1 or IL-1R1 signaling for vaccine-induced differentiation of Tc17 cells in the draining lymph nodes.** Mice were depleted of CD4^+^ T-cells and vaccinated as described in Fig. 2. Skin-draining LNs and spleens were harvested 14 to 28 days after boosting to analyze cytokine producing CD8^+^ T cells by flow cytometry. Percentage of CD8^+^ T cells expressing IFN-γ or IL-17A in Dectin-1^−/−^ and IL-1R1^−/−^ mice (**A**) and IL-6^−/−^ mice (**B**). Values are mean ± SD of 3–4 mice/group.
(TIF)

Figure S6 **Phenotypic attributes of IFN-γ^+^ Tc1 cells following vaccination.** Mice were depleted of CD4^+^ T-cells and vaccinated as described in Fig. 8. Skin-draining LNs were harvested 19 days later to analyze phenotypic attributes of KLRG-1 and TCF-1 expression among IFN-γ^+^ CD8^+^ T cells. Values are mean ± SD of 4 mice/group. Data is representative of two independent experiments.
(TIF)

## References

[1] van de Veerdonk FL, Netea MG (2010). T-cell Subsets and Antifungal Host Defenses.. Curr Fungal Infect Rep.

[2] Romani L (2011). Immunity to fungal infections.. Nat Rev Immunol.

[3] Drummond RA, Saijo S, Iwakura Y, Brown GD (2011). The role of Syk/CARD9 coupled C-type lectins in antifungal immunity.. Eur J Immunol.

[4] Brown GD (2011). Innate antifungal immunity: the key role of phagocytes.. Annu Rev Immunol.

[5] Gessner MA, Werner JL, Lilly LM, Nelson MP, Metz AE (2011). Dectin-1 dependent IL-22 contributes to early innate lung defense against Aspergillus fumigatus.. Infect Immun.

[6] Carvalho A, Cunha C, Pasqualotto AC, Pitzurra L, Denning DW (2010). Genetic variability of innate immunity impacts human susceptibility to fungal diseases.. Int J Infect Dis.

[7] van de Veerdonk FL, Kullberg BJ, van der Meer JW, Gow NA, Netea MG (2008). Host-microbe interactions: innate pattern recognition of fungal pathogens.. Curr Opin Microbiol.

[8] Dubin PJ, Kolls JK (2008). Th17 cytokines and mucosal immunity.. Immunol Rev.

[9] Gaffen SL, Hernandez-Santos N, Peterson AC (2011). IL-17 signaling in host defense against Candida albicans.. Immunol Res.

[10] Puel A, Cypowyj S, Bustamante J, Wright JF, Liu L (2011). Chronic mucocutaneous candidiasis in humans with inborn errors of interleukin-17 immunity.. Science.

[11] Werner JL, Metz AE, Horn D, Schoeb TR, Hewitt MM (2009). Requisite role for the dectin-1 beta-glucan receptor in pulmonary defense against Aspergillus fumigatus.. J Immunol.

[12] Curtis MM, Way SS (2009). Interleukin-17 in host defence against bacterial, mycobacterial and fungal pathogens.. Immunology.

[13] Wuthrich M, Gern B, Hung CY, Ersland K, Rocco N (2011). Vaccine-induced protection against 3 systemic mycoses endemic to North America requires Th17 cells in mice.. J Clin Invest.

[14] Zelante T, De Luca A, Bonifazi P, Montagnoli C, Bozza S (2007). IL-23 and the Th17 pathway promote inflammation and impair antifungal immune resistance.. Eur J Immunol.

[15] Deepe GS, Gibbons RS (2009). Interleukins 17 and 23 influence the host response to Histoplasma capsulatum.. J Infect Dis.

[16] Conti HR, Shen F, Nayyar N, Stocum E, Sun JN (2009). Th17 cells and IL-17 receptor signaling are essential for mucosal host defense against oral candidiasis.. J Exp Med.

[17] Rudner XL, Happel KI, Young EA, Shellito JE (2007). Interleukin-23 (IL-23)-IL-17 cytokine axis in murine Pneumocystis carinii infection.. Infect Immun.

[18] Huang W, Na L, Fidel PL, Schwarzenberger P (2004). Requirement of interleukin-17A for systemic anti-Candida albicans host defense in mice.. J Infect Dis.

[19] Wuthrich M, Filutowicz HI, Warner T, Deepe GS, Klein BS (2003). Vaccine immunity to pathogenic fungi overcomes the requirement for CD4 help in exogenous antigen presentation to CD8+ T cells: implications for vaccine development in immune-deficient hosts.. J Exp Med.

[20] Nigam P, Kwa S, Velu V, Amara RR (2011). Loss of IL-17-producing CD8 T cells during late chronic stage of pathogenic simian immunodeficiency virus infection.. J Immunol.

[21] Hamada H, Garcia-Hernandez Mde L, Reome JB, Misra SK, Strutt TM (2009). Tc17, a unique subset of CD8 T cells that can protect against lethal influenza challenge.. J Immunol.

[22] Yeh N, Glosson NL, Wang N, Guindon L, McKinley C (2010). Tc17 cells are capable of mediating immunity to vaccinia virus by acquisition of a cytotoxic phenotype.. J Immunol.

[23] Zhu J, Yamane H, Paul WE (2010). Differentiation of effector CD4 T cell populations (*).. Annu Rev Immunol.

[24] Korn T, Bettelli E, Oukka M, Kuchroo VK (2009). IL-17 and Th17 Cells.. Annu Rev Immunol.

[25] Chen Z, O'Shea JJ (2008). Th17 cells: a new fate for differentiating helper T cells.. Immunol Res.

[26] Zygmunt B, Veldhoen M (2011). T helper cell differentiation more than just cytokines.. Adv Immunol.

[27] Rivera A, Hohl TM, Collins N, Leiner I, Gallegos A (2011). Dectin-1 diversifies Aspergillus fumigatus-specific T cell responses by inhibiting T helper type 1 CD4 T cell differentiation.. J Exp Med.

[28] Drummond RA, Brown GD (2011). The role of Dectin-1 in the host defence against fungal infections.. Curr Opin Microbiol.

[29] Schenten D, Medzhitov R (2011). The control of adaptive immune responses by the innate immune system.. Adv Immunol.

[30] Chung Y, Chang SH, Martinez GJ, Yang XO, Nurieva R (2009). Critical regulation of early Th17 cell differentiation by interleukin-1 signaling.. Immunity.

[31] Dinarello CA (2009). Immunological and inflammatory functions of the interleukin-1 family.. Annu Rev Immunol.

[32] Sutton C, Brereton C, Keogh B, Mills KH, Lavelle EC (2006). A crucial role for interleukin (IL)-1 in the induction of IL-17-producing T cells that mediate autoimmune encephalomyelitis.. J Exp Med.

[33] Kastelein RA, Hunter CA, Cua DJ (2007). Discovery and biology of IL-23 and IL-27: related but functionally distinct regulators of inflammation.. Annu Rev Immunol.

[34] Muranski P, Borman ZA, Kerkar SP, Klebanoff CA, Ji Y (2011). Th17 cells are long lived and retain a stem cell-like molecular signature.. Immunity.

[35] Pepper M, Linehan JL, Pagan AJ, Zell T, Dileepan T (2010). Different routes of bacterial infection induce long-lived TH1 memory cells and short-lived TH17 cells.. Nat Immunol.

[36] Nanjappa SG, Heninger E, Wuthrich M, Sullivan T, Klein B (2012). Protective antifungal memory CD8(+) T cells are maintained in the absence of CD4(+) T cell help and cognate antigen in mice.. J Clin Invest.

[37] Liang SC, Long AJ, Bennett F, Whitters MJ, Karim R (2007). An IL-17F/A heterodimer protein is produced by mouse Th17 cells and induces airway neutrophil recruitment.. J Immunol.

[38] Ouyang W, Kolls JK, Zheng Y (2008). The biological functions of T helper 17 cell effector cytokines in inflammation.. Immunity.

[39] Khader SA, Bell GK, Pearl JE, Fountain JJ, Rangel-Moreno J (2007). IL-23 and IL-17 in the establishment of protective pulmonary CD4+ T cell responses after vaccination and during Mycobacterium tuberculosis challenge.. Nat immunol.

[40] Lin Y, Ritchea S, Logar A, Slight S, Messmer M (2009). Interleukin-17 is required for T helper 1 cell immunity and host resistance to the intracellular pathogen Francisella tularensis.. Immunity.

[41] Hu W, Troutman TD, Edukulla R, Pasare C (2011). Priming microenvironments dictate cytokine requirements for T helper 17 cell lineage commitment.. Immunity.

[42] Kim CH (2009). Migration and function of Th17 cells.. Inflamm Allergy Drug Targets.

[43] Chen K, McAleer JP, Lin Y, Paterson DL, Zheng M (2011). Th17 cells mediate clade-specific, serotype-independent mucosal immunity.. Immunity.

[44] Iwakura Y, Nakae S, Saijo S, Ishigame H (2008). The roles of IL-17A in inflammatory immune responses and host defense against pathogens.. Immunol Rev.

[45] Taylor PR, Tsoni SV, Willment JA, Dennehy KM, Rosas M (2007). Dectin-1 is required for beta-glucan recognition and control of fungal infection.. Nat Immunol.

[46] Saijo S, Fujikado N, Furuta T, Chung SH, Kotaki H (2007). Dectin-1 is required for host defense against Pneumocystis carinii but not against Candida albicans.. Nat Immunol.

[47] Gringhuis SI, den Dunnen J, Litjens M, van der Vlist M, Wevers B (2009). Dectin-1 directs T helper cell differentiation by controlling noncanonical NF-kappaB activation through Raf-1 and Syk.. Nat Immunol.

[48] Weber A, Wasiliew P, Kracht M (2010). Interleukin-1 (IL-1) pathway.. Sci Signal.

[49] Romagnani S, Maggi E, Liotta F, Cosmi L, Annunziato F (2009). Properties and origin of human Th17 cells.. Mol Immunol.

[50] Martin-Orozco N, Muranski P, Chung Y, Yang XO, Yamazaki T (2009). T helper 17 cells promote cytotoxic T cell activation in tumor immunity.. Immunity.

[51] Groom JR, Luster AD (2011). CXCR3 ligands: redundant, collaborative and antagonistic functions.. Immunol Cell Biol.

[52] Cox CA, Shi G, Yin H, Vistica BP, Wawrousek EF (2008). Both Th1 and Th17 are immunopathogenic but differ in other key biological activities.. J Immunol.

[53] Khanolkar A, Badovinac VP, Harty JT (2007). CD8 T cell memory development: CD4 T cell help is appreciated.. Immunol Res.

[54] Hirota K, Duarte JH, Veldhoen M, Hornsby E, Li Y (2011). Fate mapping of IL-17-producing T cells in inflammatory responses.. Nat Immunol.

[55] Onami TM, Harrington LE, Williams MA, Galvan M, Larsen CP (2002). Dynamic regulation of T cell immunity by CD43.. J Immunol.

[56] Mody PD, Cannon JL, Bandukwala HS, Blaine KM, Schilling AB (2007). Signaling through CD43 regulates CD4 T-cell trafficking.. Blood.

[57] Clark MC, Baum LG (2012). T cells modulate glycans on CD43 and CD45 during development and activation, signal regulation, and survival.. Ann N Y Acad Sci.

[58] Hikono H, Kohlmeier JE, Takamura S, Wittmer ST, Roberts AD (2007). Activation phenotype, rather than central- or effector-memory phenotype, predicts the recall efficacy of memory CD8+ T cells.. J Exp Med.

[59] Zhou X, Yu S, Zhao DM, Harty JT, Badovinac VP (2010). Differentiation and persistence of memory CD8(+) T cells depend on T cell factor 1.. Immunity.

[60] Joshi NS, Cui W, Chandele A, Lee HK, Urso DR (2007). Inflammation directs memory precursor and short-lived effector CD8(+) T cell fates via the graded expression of T-bet transcription factor.. Immunity.

[61] Yu Q, Sharma A, Ghosh A, Sen JM (2011). T cell factor-1 negatively regulates expression of IL-17 family of cytokines and protects mice from experimental autoimmune encephalomyelitis.. J Immunol.

[62] Ma J, Wang R, Fang X, Ding Y, Sun Z (2011). Critical role of TCF-1 in repression of the IL-17 gene.. PloS one.

[63] Hardison SE, Wozniak KL, Kolls JK, Wormley FL (2010). Interleukin-17 is not required for classical macrophage activation in a pulmonary mouse model of Cryptococcus neoformans infection.. Infect Immun.

[64] Jabbari A, Harty JT (2006). Simultaneous assessment of antigen-stimulated cytokine production and memory subset composition of memory CD8 T cells.. J Immunol Methods.

