# Correction: Tc17 Cells Mediate Vaccine Immunity against Lethal Fungal Pneumonia in Immune Deficient Hosts Lacking CD4^+^ T Cells

**DOI:** 10.1371/journal.ppat.1004148

**Published:** 2014-04-28

**Authors:** The PLOS Pathogens Staff

There are errors in Figure 6 and Supplementary Figures S4 and S5.

**Figure 6:**
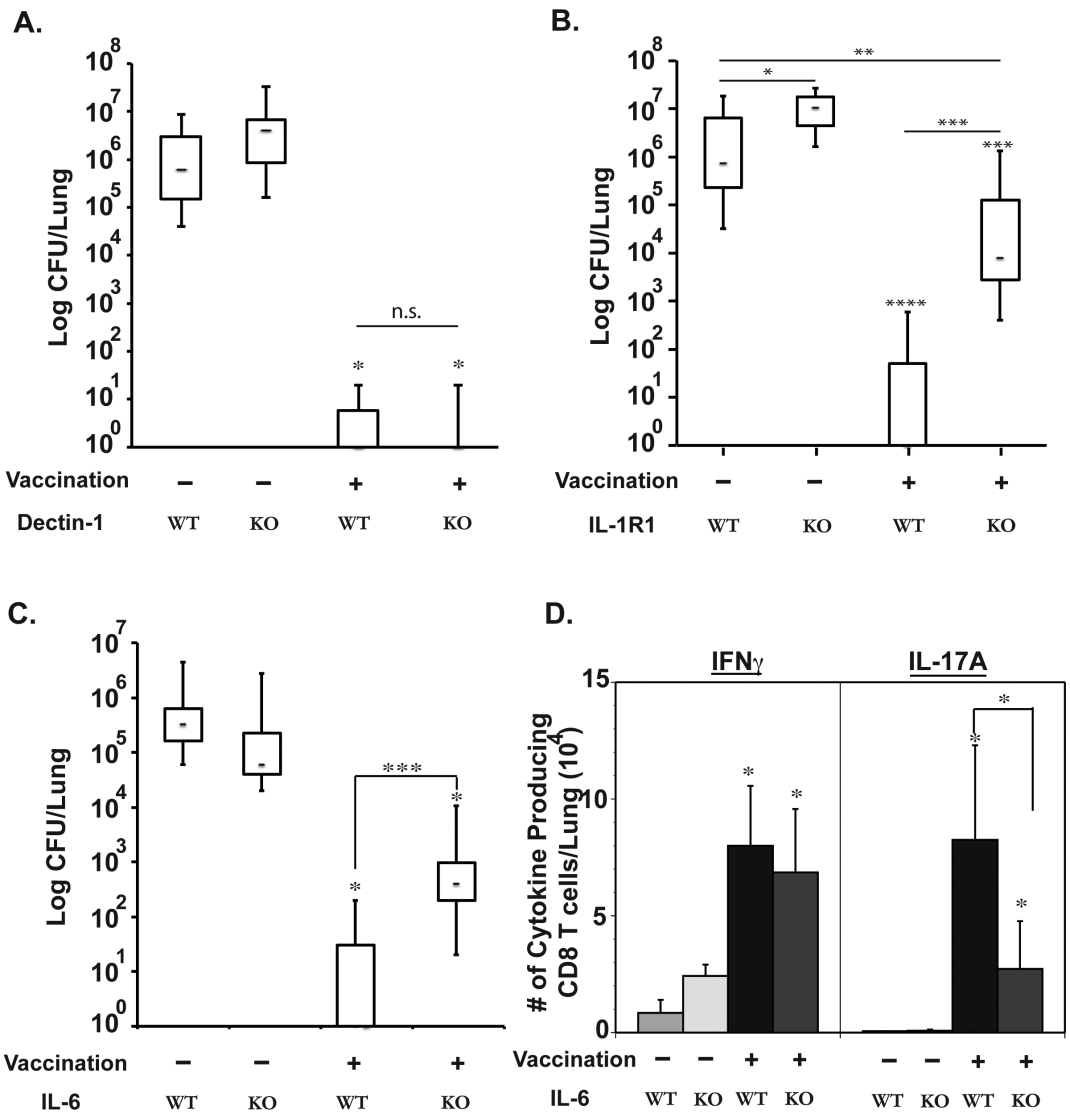
Roles of Dectin-1, IL-1R1 and IL-6 in the induction of Tc17 cells and anti-fungal vaccine immunity. Mice were depleted of CD4^+^ T cells, vaccinated and challenged as described in Fig. 2. **A, B and C**. Lung CFU values (in box and whisker plots). Three weeks post-challenge in Dectin-1^−/−^ and WT controls (N  =  7–10/group) (**A**); 16 days post-challenge in IL-1R1^−/−^ mice and WT controls (N  =  7–13/ group) (**B**); and day11 post-challenge in IL-6^−/−^ mice and WT controls (N  =  5–11) (**C**). **D.** Number of cytokine-producing cells recruited to the lungs 4 days after challenge of IL-6−/− and WT control mice, as measured by flow cytometry. *, p<0.01 vs. unvaccinated controls unless otherwise indicated. The corrected versions of Supplementary Figures S4 and S5 can be downloaded here. The legends are changed and provided below.

The authors would like to correct the phenotype/function observed for Tc17 cells in IL1-R1 knockout mice. They recently learned that the mice were heterozygotes and not homozygotes. The corrected figures show the data correction for IL1-R1-/- homozygote mice in terms of their blunted vaccine resistance in Figure 6B; the blunted number of CD8 T cells in the spleen that make IL-17 and other cytokines in Figure S4B; and the reduced percentage of IL-17 producing CD8 T cells in the draining lymph nodes of vaccinated mice in Figure S5A in the absence of IL1-R1, as compared to wild-type mice.

The corrected version of Figure 6 can be seen here. The legend for Figure 6B is changed and provided below.

The corrected versions of Supplementary Figure S4 and S5 can be downloaded here. The legends are changed and provided below.

## Supporting Information

Figure S4 **Roles of Dectin-1 and IL-1R1 signaling for vaccine-induced Tc17 cells.** Groups of wild-type and Dectin-1^−/−^ mice were depleted of CD4^+^ T-cells and vaccinated as described in Figure 2. Two weeks after the boost, mice were challenged intratracheally with 2×10^3^ cfu of wild-type yeast. Four days later, mice were sacrificed; lungs were harvested and analyzed for intracellular cytokine staining by flow cytometry. Total number of cytokine producing CD8^+^ T cells in Dectin-1^−/−^ (A) and IL-1R1^−/−^ (B) and wild-type mice. Values are mean ± SD of 5–6 mice/group.

Figure S5 **Non-redundant role of IL-6 and IL-1R1 signaling, but not Dectin-1 for vaccine-induced differentiation of Tc17 cells in the draining lymph nodes.** Mice were depleted of CD4^+^ T-cells and vaccinated as described in Figure 2. Skin-draining LNs and spleens were harvested 14 to 28 days after boosting to analyze cytokine producing CD8^+^ T cells by flow cytometry. Percentage of CD8^+^ T cells expressing IFN-γ or IL-17A in Dectin-1^−/−^ and IL-1R1^−/−^ mice (A) and IL-6^−/−^ mice (B). Values are mean ± SD of 3–4 mice/group.
