# Supplementary figures and images for: Tc17 Cells Mediate Vaccine Immunity against Lethal Fungal Pneumonia in Immune Deficient Hosts Lacking CD4+ T Cells
Source: PLoS Pathog. 2012 Jul 19;8(7):e1002771. doi: 10.1371/journal.ppat.1002771 (PMC3400565; doi:10.1371/journal.ppat.1002771)

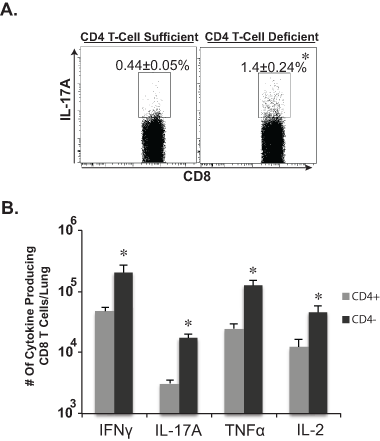

Supplement: Figure S1 — Tc17 cells are recalled in the lung in the absence of CD4+ T cells upon Histoplasma capsulatum infection. Mice were vaccinated s.c. with ∼106 cfu of H. capsulatum yeast. A weekly dose of 100 µg GK1.5 mAb was used to deplete CD4+ T cells. After 8–9 wks, mice were challenged intratracheally with sublethal dose of 2×10^5^ cfu and 4 days later the lungs were harvested to analyze cytokine producing CD8+ T cells by flow cytometry. A. Dot plot shows percent CD8+ T cells expressing IL-17A in CD4+ T-cell sufficient and depleted mice. B. Total number of cytokine-producing CD8+ T cells/lung in CD4+ T-cell sufficient and depleted groups. Values are mean ± SD of 3–5 mice/group. *, p<0.05. (TIF) [file ppat.1002771.s001.tif]

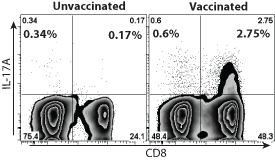

Supplement: Figure S2 — IL-17A producing cells in the lung of vaccinated mice after infection. Mice were depleted of CD4+ T cells, vaccinated and intratracheally infected as described in Fig. 1 and 2. Lung cells were harvested, re-stimulated and assessed for IL-17A producing cells by flow cytometry. The numbers in the plot indicate the percent of cells among lymphocyte-gated total lung cells. (TIF) [file ppat.1002771.s002.tif]

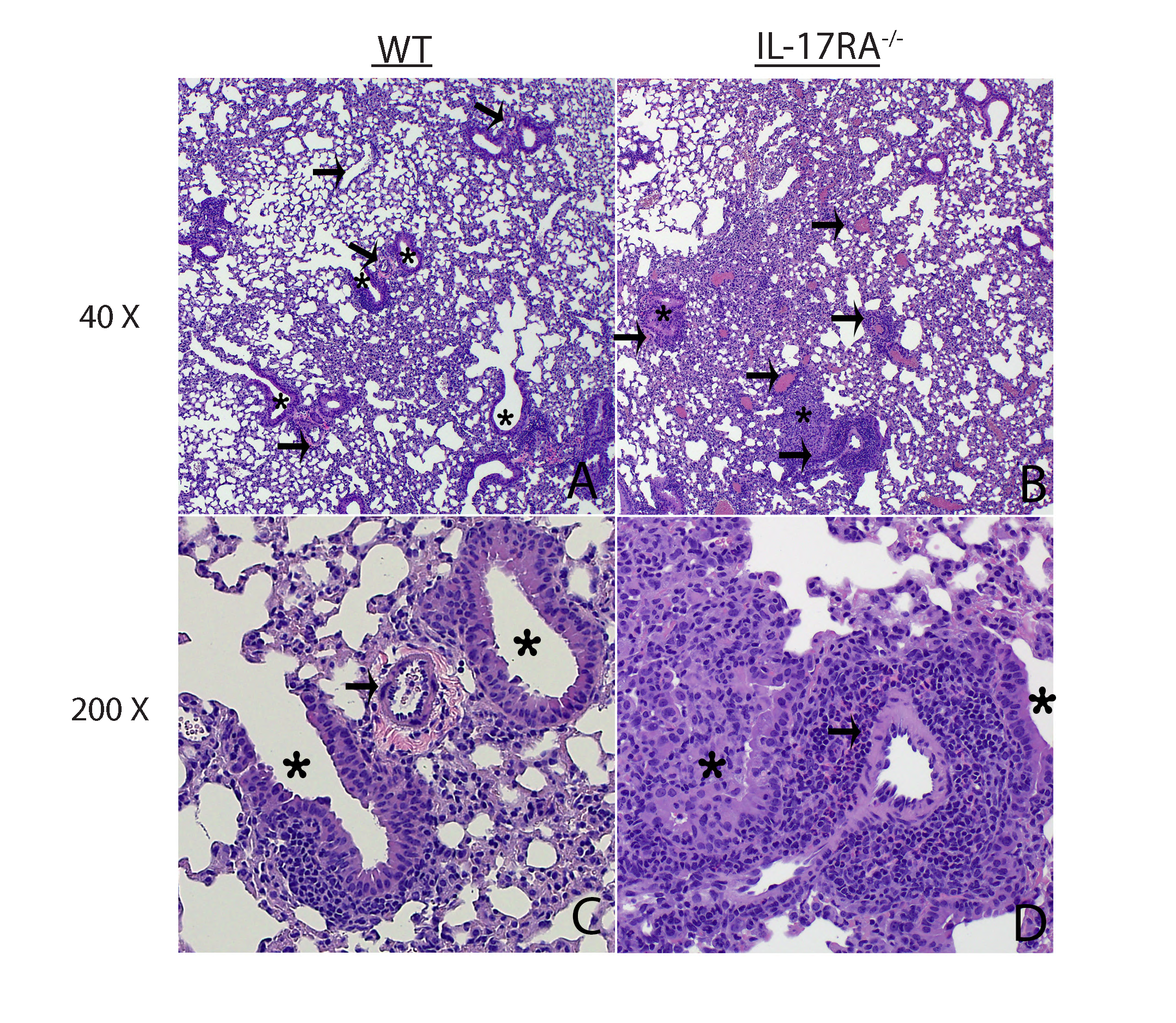

Supplement: Figure S3 — Pulmonary inflammation in vaccinated mice in the absence of IL-17A signaling following infection. Mice were depleted of CD4+ T cells, vaccinated and intratracheally infected as described in Fig. 1 and 2. Lung tissues were collected and stored in 10% neutral buffered formalin. Lung tissue sections were taken and stained with H&E for histopathology studies. Upper panels images are at 40× magnification; lower panels are at 200×. Vaccinated wild-type mice have mostly a peribronchiolar pattern of inflammation (asterisks), while the knockout mice exhibit mostly a perivascular pattern (arrows). Nodular bronchocentric foci of inflammation with bronchiolar exudate are present in the knockout mice (B), but these foci of inflammation are rare in wild type mice (A). The peribronchiolar and perivascular infiltrates in both wild-type (C) and knock out (D) mice are composed of lymphocytes, plasma cells, few intact neutrophils, and rare histiocytes. In contrast, the bronchiolar exudate (asterisks) and peribronchiolar nodular infiltrates are primarily histocytic with fewer neutrophils and lymphocytes (D). (TIF) [file ppat.1002771.s003.tif]

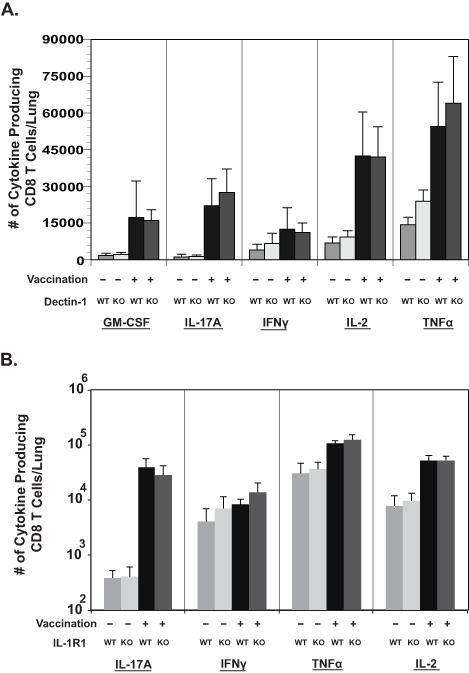

Supplement: Figure S4 — Dispensability of Dectin-1 and IL-1R1 signaling for vaccine-induced Tc17 cells recruited to the lung. Groups of wild-type and Dectin-1−/− mice were depleted of CD4+ T-cells and vaccinated as described in Fig. 2. Two weeks after the boost, mice were challenged intratracheally with 2×10^3^ cfu of wild-type yeast. Four days later, mice were sacrificed; lungs were harvested and analyzed for intracellular cytokine staining by flow cytometry. Total number of cytokine producing CD8+ T cells in Dectin-1−/− (A) and IL-1R1−/− (B) and wild-type mice. Values are mean ± SD of 5–6 mice/group. (TIF) [file ppat.1002771.s004.tif]

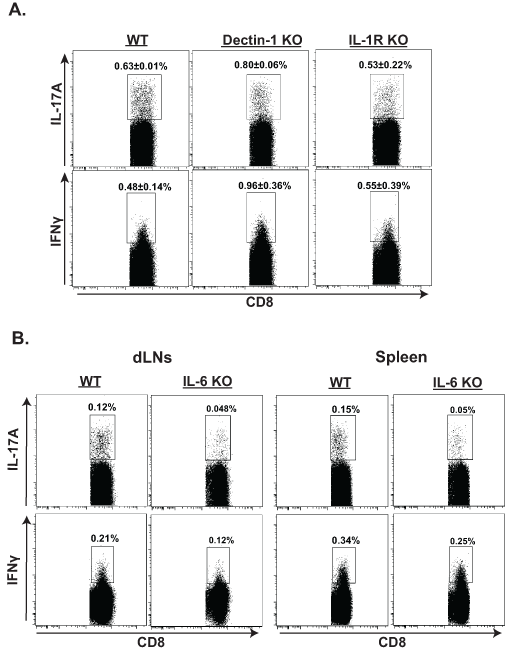

Supplement: Figure S5 — Non-redundant role of IL-6, but not Dectin-1 or IL-1R1 signaling for vaccine-induced differentiation of Tc17 cells in the draining lymph nodes. Mice were depleted of CD4+ T-cells and vaccinated as described in Fig. 2. Skin-draining LNs and spleens were harvested 14 to 28 days after boosting to analyze cytokine producing CD8+ T cells by flow cytometry. Percentage of CD8+ T cells expressing IFN-γ or IL-17A in Dectin-1−/− and IL-1R1−/− mice (A) and IL-6−/− mice (B). Values are mean ± SD of 3–4 mice/group. (TIF) [file ppat.1002771.s005.tif]

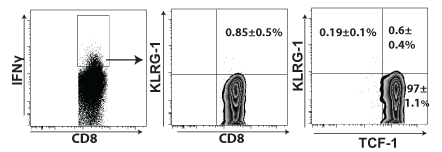

Supplement: Figure S6 — Phenotypic attributes of IFN-γ+ Tc1 cells following vaccination. Mice were depleted of CD4+ T-cells and vaccinated as described in Fig. 8. Skin-draining LNs were harvested 19 days later to analyze phenotypic attributes of KLRG-1 and TCF-1 expression among IFN-γ+ CD8+ T cells. Values are mean ± SD of 4 mice/group. Data is representative of two independent experiments. (TIF) [file ppat.1002771.s006.tif]
