# Supplementary figures and images for: Correction: Tc17 Cells Mediate Vaccine Immunity against Lethal Fungal Pneumonia in Immune Deficient Hosts Lacking CD4+ T Cells
Source: PLoS Pathog. 2014 Apr 28;10(4):e1004148. doi: 10.1371/journal.ppat.1004148 (PMC4002485; doi:10.1371/journal.ppat.1004148)

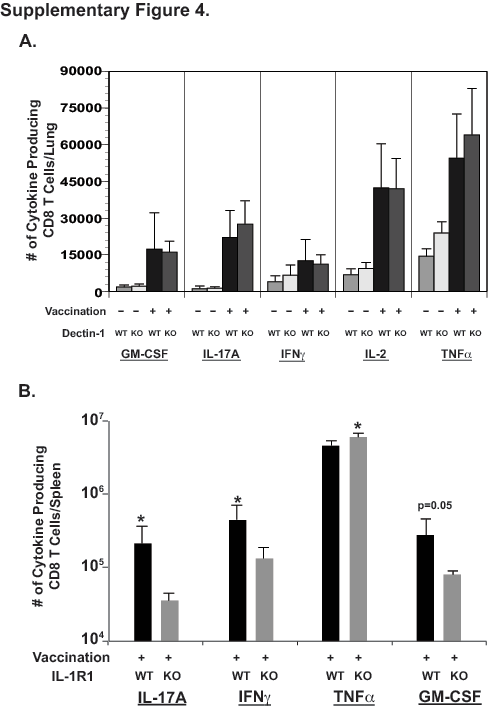

Supplement: Figure S4 — Roles of Dectin-1 and IL-1R1 signaling for vaccine-induced Tc17 cells. Groups of wild-type and Dectin-1−/− mice were depleted of CD4+ T-cells and vaccinated as described in Figure 2. Two weeks after the boost, mice were challenged intratracheally with 2×10^3^ cfu of wild-type yeast. Four days later, mice were sacrificed; lungs were harvested and analyzed for intracellular cytokine staining by flow cytometry. Total number of cytokine producing CD8+ T cells in Dectin-1−/− (A) and IL-1R1−/− (B) and wild-type mice. Values are mean ± SD of 5–6 mice/group. [file ppat.1004148.s001.tif]

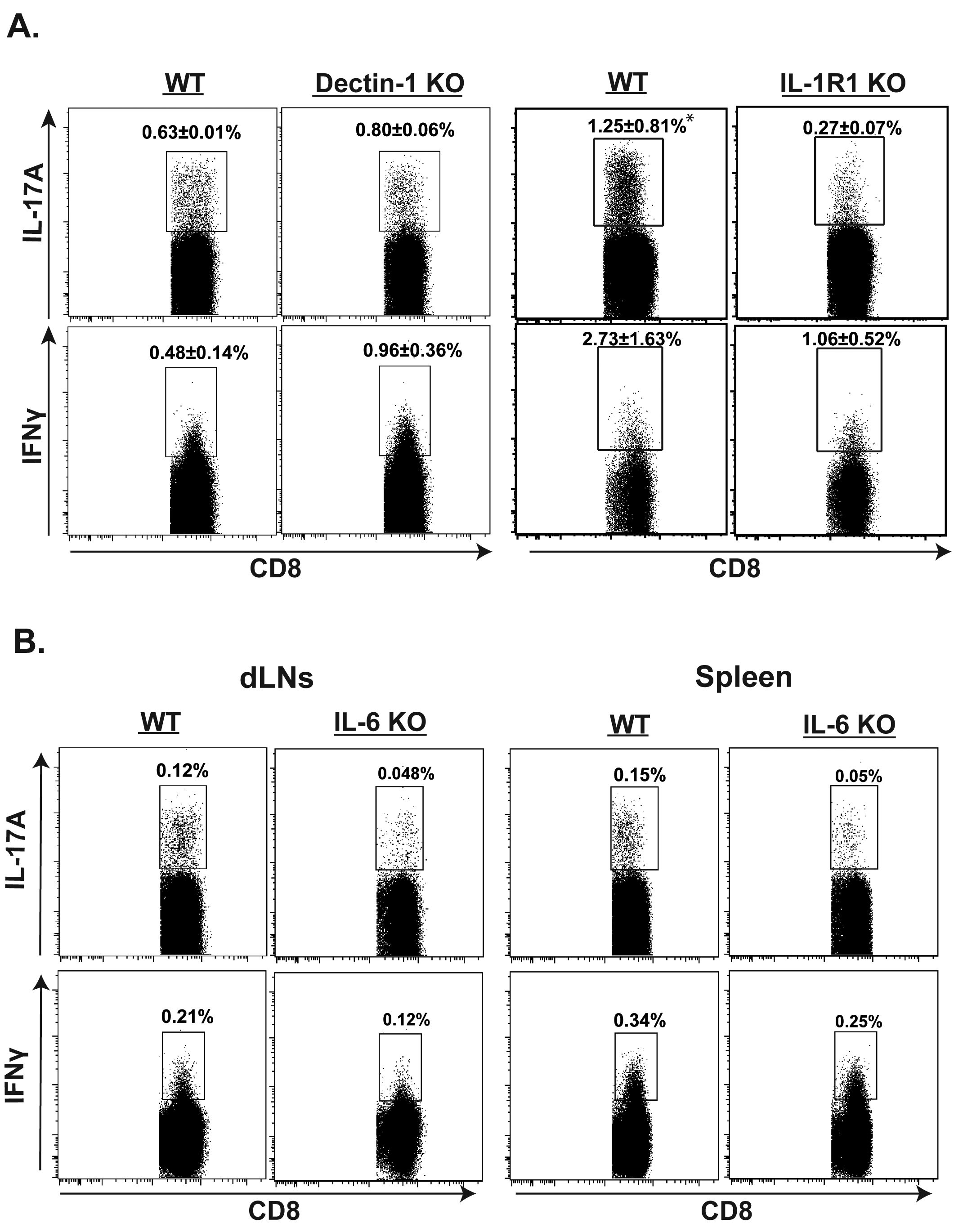

Supplement: Figure S5 — Non-redundant role of IL-6 and IL-1R1 signaling, but not Dectin-1 for vaccine-induced differentiation of Tc17 cells in the draining lymph nodes. Mice were depleted of CD4+ T-cells and vaccinated as described in Figure 2. Skin-draining LNs and spleens were harvested 14 to 28 days after boosting to analyze cytokine producing CD8+ T cells by flow cytometry. Percentage of CD8+ T cells expressing IFN-γ or IL-17A in Dectin-1−/− and IL-1R1−/− mice (A) and IL-6−/− mice (B). Values are mean ± SD of 3–4 mice/group. [file ppat.1004148.s002.tif]
